# Mental health concerns precede quits: shifts in the work discourse during the Covid-19 pandemic and great resignation

**DOI:** 10.1140/epjds/s13688-023-00417-2

**Published:** 2023-10-12

**Authors:** R. Maria del Rio-Chanona, Alejandro Hermida-Carrillo, Melody Sepahpour-Fard, Luning Sun, Renata Topinkova, Ljubica Nedelkoska

**Affiliations:** 1 Complexity Science Hub, Vienna, Austria; 2 Growth Lab, Harvard Kennedy School, Harvard University, Cambridge, MA USA; 3 LMU Munich School of Management, Munich, Germany; 4 Science Foundation Ireland Centre for Research Training in Foundations of Data Science, Limerick, Ireland; 5 Department of Mathematics and Statistics (MACSI), University of Limerick, Limerick, Ireland; 6 The Psychometrics Centre, University of Cambridge, Cambridge, UK; 7 Department of Sociology, LMU Munich, Munich, Germany

**Keywords:** Mental health, Quit, Great Resignation, Topic modelling, Labor market, COVID-19

## Abstract

To study the causes of the 2021 Great Resignation, we use text analysis and investigate the changes in work- and quit-related posts between 2018 and 2021 on Reddit. We find that the Reddit discourse evolution resembles the dynamics of the U.S. quit and layoff rates. Furthermore, when the COVID-19 pandemic started, conversations related to working from home, switching jobs, work-related distress, and mental health increased, while discussions on commuting or moving for a job decreased. We distinguish between general work-related and specific quit-related discourse changes using a difference-in-differences method. Our main finding is that mental health and work-related distress topics disproportionally increased among quit-related posts since the onset of the pandemic, likely contributing to the quits of the Great Resignation. Along with better labor market conditions, some relief came beginning-to-mid-2021 when these concerns decreased. Our study underscores the importance of having access to data from online forums, such as Reddit, to study emerging economic phenomena in real time, providing a valuable supplement to traditional labor market surveys and administrative data.

**Supplementary Information:**

The online version contains supplementary material available at 10.1140/epjds/s13688-023-00417-2.

## Introduction

In April 2021, 3.9 million workers quit their jobs in the U.S., the highest recorded quit rate in at least the last 30 years (Davis and Haltiwanger [15], Bureau of Labor Statistics [12]). During 2021, the U.S. quit rates remained high (Bureau of Labor Statistics [12]) and high quit rates were also reported by the media in other countries (Horowitz [30], Gupta [24], Khan [34]). This phenomenon has been named the “Great Resignation” and received considerable attention from news outlets (Casselman [13], Rosalsky [59], Donegan [20], Romm [58]). Given that people deeply care about employment events (Alan [3]), the record high quit rate raises concerns about worker well-being; and since the costs of losing workers are high (O’Connell and Kung [48]), the Great Resignation poses a big challenge for recovering businesses.

In spite of the relevance of this topic, there is a dearth of academic literature studying this phenomenon. A recent pre-print reported survey results that suggest the pandemic caused some workers to drop out of the labor force permanently (Barrero et al. [6]). Besides this work, there are some newspaper and magazine articles empirically covering the topic. Economist Paul Krugman analyzed U.S. labor market data and argued in the New York Times (Krugman [36]) that the rise in quits was less driven by labor force drop-outs and more due to people switching jobs and starting new businesses. Cook [14] and Sull et al. [65] analyzed private data sets of employee profiles and records and identified mental health, burn-out, postponed resignations, and toxic work environments as possible explanations. Although these newspaper and magazine articles present compelling causes of the Great Resignation, there is no consensus on the extent to which it was driven by job switching, self-employment, labor force reductions, burn-out and/or mental health concerns.

While the academic literature on the Great Resignation is scarce, there is an extensive literature in management science and labor economics studying quit behaviour from which we can draw upon. We know that people usually quit their jobs to pursue new, likely better ones (Hall and Lazear [25], Lazear and Spletzer [38], Lee et al. [39]). This leads to a pro-cyclicality in quit rates: quits peak in economic expansions, when job openings are plentiful and of higher quality, and plummet in economic recessions, when job openings are scarce (Davis and Haltiwanger [15]). Most likely, the pro-cyclicality of quit rates is partially the story behind the 2021 Great Resignation. After a historically sharp disruption of the labor market in the first two quarters of 2020, the economy quickly recovered, creating record numbers of job openings, increasing nominal wages (Furman and Powell III [22]), inducing job switches (Parker and Horowitz [49]) and hence a high number of quits. In this sense, the pro-cyclical behaviour of job openings are *pull factors* that increase the quit rate after a recession.

However, it is unlikely that the pro-cyclicality of the quit rates explains the full extent of the Great Resignation. First, the increase in the labor market tightness (i.e., job openings to unemployed workers ratio) suggests a significant decline in matching efficiency between job-seekers and job openings (Rodgers and Kassens [56]). Second, the COVID-19 pandemic unleashed a chain of *push factors* (i.e., factors that affected people’s work experience and may have incentivized them to quit) not present in a typical recession that presumably contributed to the rising number of quits. In addition to the immediate consequences of personal exposure to the SARS-CoV-2 virus, workers had to cope with school closures and online schooling, caring after sick family and friends, and workplace burnout in sectors seen as essential. As a combined effect of the pandemic’s aftermath, working people experienced psychological pressures both at the workplace (Sull et al. [65], Cook [14]) and at home (Donegan [20]), and may have driven part of the recorded increases in anxiety (Ashokkumar and Pennebaker [5]) and a worsening of the population’s mental health state (Xiong et al. [70]). From a management science perspective, the COVID-19 pandemic was a shock (i.e., a jarring event), and as such, may have triggered people to think about quitting (Lee and Mitchell [40], Morgeson et al. [45], Akkermans et al. [2]). These cognition processes have been labelled “pandemic epiphanies” by the media (Rosalsky [59]) and are also push factors the pandemic released.

Although the above mentioned push factors likely contributed to the rise in quits during the Great Resignation, it is difficult to identify these contributions through traditional economic data for several reasons. First, traditional labor market surveys and administrative data seldom include measures of mental health or other factors that may influence people’s willingness to work. Second, some of the above cited articles (Parker and Horowitz [49], Sull et al. [65]) rely on snapshot data from a single survey, making it difficult to capture pre- and post- pandemic changes. Third, surveys may limit participants’ expression by having a set of predefined questions and, since employment can be a sensitive topic, the responses may be biased (Tourangeau et al. [66]). Fourth, surveys and administrative data are costly and time-consuming to collect. The latter may be one of the main reasons why the Great Resignation is still under-explored in the academic literature.

Data from Reddit, an online platform that allows users to discuss and share experiences around topics of interest called subreddits, poses an alternative for studying the Great Resignation overcoming the above mentioned challenges. Reddit has the advantage that posts are semi-anonymous and of unrestricted length, allowing individuals to express themselves freely and in detail. Furthermore, data can be collected in real-time through an API (Baumgartner et al. [7]). Recent works have also used Reddit to study a diverse range of socio-economic phenomena. Semenova and Winkler [62] and Lucchini et al. [42] studied the 2021 increase in GameStop’s shares through the subreddit ‘r/WallStreetBets’. Sepahpour-Fard and Quayle [63] studied parenting concerns while Waller and Anderson [68] studied polarization on Reddit and Perry and DeDeo [51] extremist ideologies.

The goal of this study is to uncover shifts in the work discourse after the COVID-19 pandemic and the causes underpinning the surge in quit rates in the U.S. in 2021 (i.e. the causes of the Great Resignation). Our first research question is: How did the work discourse change since the onset of the pandemic and during the Great Resignation? One could expect that people talked more about remote work, job switching and health and mental health concerns. Our second research question is whether, relative to the general work discourse, some of these topics increased more among users thinking about quitting? Given that quit-cognitions are a strong predictor of quitting (Rubenstein et al. [61]), by answering this last question we can identify possible drivers of the 2021 record high quit rate.

To answer these questions we analyze the content of work- and quit-related posts on Reddit from a majority of U.S.-based users spanning the period from 2018 to 2021. We use topic modeling — a widely used text analysis method (Kennedy et al. [33]). We find that Reddit discourse captures known work-related changes that the pandemic brought, namely the rise of remote work and decline in commuting. Furthermore, switching jobs, work-related distress and mental health topics increased their prevalence after the start of the pandemic. These findings validate our method, showing that the work discourse is inline with studies showing that the COVID-19 pandemic worsened the mental health of the general population across the world (see Xiong et al. for a review) and with the discussions relating the Great Resignation with job switching and mental health concerns (Krugman [35], Cook [14], Sull et al. [65]).

Our main finding is that the pandemic exacerbated the already growing mental health concerns among workers, and we show that such concerns became disproportionately present in the discourse of quit-related posts since the onset of the pandemic. Furthermore, posts about mental health and work-related distress are more likely to involve quitting. We argue that, while the increase in job vacancies and job switching were factors present in previous economic recovery periods, the COVID-19 pandemic unleashed forces leading to quit behavior, such as mental health concerns, that were absent in previous recoveries. These additional factors could help explain the unusually high rates of quitting in 2021.

To complement the above result we ask to what extent was the contribution of mental health concerns to the increase in the quit discourse driven by (a) an increase in mental health concerns or (b) an increase in the strength of the relationship between mental health concerns and quits. That is, people may have been more likely to mention quitting when talking about mental health after the pandemic, regardless of whether more people talked about mental health or not. We use a multiple regression analysis to answer this question and find that the relationship between mental health concerns and quits remained roughly constant. This means that the onset of the pandemic did not change the character of the relationship between push factors and quit considerations, but it elevated the prevalence of phenomena such as mental health and work-related distress, which in return led to more quits.

## Data and methods

### Extraction and filtering of Reddit posts

We extract submissions posted between January 2016 and December 2021 (inclusive) in the ‘r/jobs’ subreddit, a popular work-related subreddit targeted to a general working-age audience. We access the data via Pushshift API (Baumgartner et al. [7]). As we discuss in Additional file 1 S3, ‘r/jobs’ has a posting history going a few years back before the pandemic, and is therefore better suited to study the causes of the Great Resignation than subreddits such as ‘r/antiwork’ which was heavily covered by the news when discussing the Great Resignation (Rogers [57]).

For each post, we have, among other data, the username of the author, the date when it was posted, title, text content of the post, and flair (i.e., a predefined tag users can add to specify a post’s topic). We remove all posts that were flaired as spam, scams, removals or moderator or bot posts as well as those with no title and text content. Furthermore, we focus only on years 2018-2021, the period when the majority of the posts were made. This leaves 269,647 ‘r/jobs’ posts. To avoid biasing our study towards authors that post a lot but may not represent an average user, we remove all posts that correspond to authors who have more than 10 posts in the subreddit.

### Uncovering the characteristics of the Reddit population

To contextualize our findings, it is important to understand demographic characteristics of the population of ‘r/jobs’. Measuring these characteristics is challenging; Reddit users are not geolocated, often use gender-neutral pseudonyms, and rarely portray personal information in their profile. Nonetheless, we examine the ‘r/jobs’ population in two ways. First, we use the neural-embeddings provided by Waller and Anderson [68] that characterize the populations of popular subreddits in terms of age, gender and U.S. political partisanship. Second, we examine the posting history of a subsample of ‘r/jobs’ users to infer their demographic characteristics using an approach similar to von Hippel and Cann [67]. Specifically, we randomly select the authors of 200 posts before and 200 posts after March 1st 2020, i.e., the date we use as the onset of the pandemic, from our ‘r/jobs’ post sample. With the help of a research assistant, we hand-code self-disclosed demographics in the Reddit posting history of each author (e.g., a user writing “f/20” in a post).

### Text pre-processing

Our text pre-processing workflow follows the recommendations by Hickman et al. [28] for both closed and open vocabulary approaches. We first concatenate the title and body of the post, referred from here onwards as the text. We lowercase all text and remove special characters and sequences such as quotes, newlines, parenthesis, etc. We unravel the acronyms “pto” and “wfh” to “paid time off” and “work from home”; we remove the acronym “tldr” (too long didn’t read). We homogenize different ways to refer to the COVID-19 pandemic to ‘covid’, e.g., we replace ‘coronavirus’ and ‘covid-19’ with ‘covid’ (we do not replace ‘pandemic’). For handling negation, as our method is dictionary-based, we create n-grams following negation words using part-of-speech tagging, i.e., if a negation word is followed by a noun, an adjective, or a verb within a three-word window, an underscore will be added between the corresponding words to be ignored by the emotion lexicon. For example, the phrase ‘not happy’ will become ‘not_happy’. This way, ‘happy’ is not classified as a positive word as it would be the case without negation handling.

For topic modelling, we take the following additional steps. We remove a set of common stopwords (i.e., non-content words such as “the”, “do”, or “throughout”) using Scikit-learn’s (Pedregosa et al. [50]) list of English stopwords to which we add “ai”, “im”, “m”, “s”, “ve”, “w”, “d”, “ive”, “id”, “itll”. Then, we form bigrams or trigrams from ordered set of words that commonly appear together, and lemmatize unigrams. After n-gram formation, we delete tokens which are overly common (“job”, “like”, “just”), boilerplate (“andor”, “http”, “amp”), or idiosyncratic to Reddit (“long_post”, “sorry_long”, “rjobs”, “hey_guy”). We then create the final document-feature-matrix (dfm) restricting the used terms to those appearing in a minimum of .5% and a maximum of 99% of all texts.

### Labelling posts and building a control group

Our unit of analysis is time-stamped Reddit posts from 2018-2021, where a post is an original submission without follow-up comments. We aggregate time stamps into monthly and quarterly intervals to have a significant amount of posts for each period. To compare the U.S. quit and layoff rates to r/jobs data, we label posts as quit- or fired-related by using keywords. We use the following keywords to identify fired-related posts: cann*, la[i,y]*off, laid (me) off, ax[e,i]*, fir[e,i]*, terminat*, sack([e,i]*), job(*)loss, redundan*, let* (me) go, lost (my, the, a) job, pink(*)slip, where * denotes any character, [ ] denotes match one out of several characters, and () denotes optional character sequence.^1^ To identify the quit-related posts, we use the following keywords: resign*, quit*, leav* (my, the, a) job, left (my, the, a) job, bow* out, (week*, my, a, the) notice, switch* (*) job, chang* (*) job, look* for (*) new job.

In the subsequent analyses, we use keywords to label posts as quit- and nonquit- related. To do this, we go through the text of a post and search for quit-related keywords. If we find at least one keyword related to quitting in the post, we label said post as quit-related, otherwise, we label it as nonquit-related. We find 26,016 and 172,065 quit- and nonquit- related posts respectively.

We build a control group of posts that can help discern how the content of quit-related posts has changed since the onset of the pandemic not only relative to the pre-pandemic levels, but also relative to posts that are not quit-related. Initially, we considered building the control group from a subsample of other subreddits. However, this method appeared to yield a poor control group, due to the dominance of topics that have little to do with people’s work, and, likely, due to the different demographic structure of the contributors. Hence, we build the control group using the nonquit-related posts of ‘r/jobs’. From this sample, we exclude all posts made by authors that had quit-related posts. We then take a random sample of $m_{y}$, where $m_{y}$ is the number of quit-related posts for year *y* (4996, 6860, 6080, and 8080 for 2018 to 2021 respectively). Therefore, in each year, the control group has the same size as the treatment group. This procedure leads to a sample of roughly 52,000 posts, half quit related and half control group.

### Structural topic modelling

We use Structural Topic Model (STM), an unsupervised clustering method aimed to discover latent topics from texts automatically (Blei [9]). In these models, topics are a distribution over words and documents are a distribution over topics. The *prevalence* of a topic in a document is the probability that a document belongs to a said topic. The basic assumption is that words which co-occur in documents (Reddit posts in our case) discuss the same subject. Moreover, documents may have a mixture of topics, in this case the model computes the prevalence of each topic in a per-document basis. Structural Topic Models have two main advantages: First, they allows for topic correlations, i.e., to account for the fact that some combinations of topics are more likely to co-occur. Second, these models allow within-document topic prevalence to differ across metadata such as the time in which the text was written.

We use the packages *stm* (Roberts et al. [54]) and *quanteda* (Benoit et al. [8]) in the statistical environment *R* (R Core Team [53]) to fit the Structural Topic Models. For the analysis in the main text, we report results using only one Structural Topic Model. However, in Additional file 1 S5 we report robustness checks for Structural Topic Models fitted using parameter variations.

We fit the main Structural Topic Model using the following two-period equation

1 $$ y_{it} = \alpha + \beta _{1}T_{t} + \beta _{2}Q_{it}+\epsilon _{it} , $$

where $\beta _{1}$ is the vector with the coefficients of interest, which measure changes in prevalence in time for each topic; $T_{t}$ is a dummy variable which takes a value of 0 until February 2020, and a value of 1 starting in March 2020; $Q_{it}$ is a variable controlling for the structure of the text (quit- vs. nonquit- related posts) which takes a value of 1 for posts that are quit-related and 0 for posts in the control group; *α* is the constant and $\epsilon _{it}$ the error term. Equation (1) allows us to study the change in the work discourse after the onset of the COVID-19 pandemic.

To determine the number of topics *K*, we fit the model varying *K* in increments of 5 and measure exclusivity, semantic coherence, held out likelihood and residuals for each value of *K* (see Fig. S1 in Additional file 1). We follow Hofstra et al. [29] and balance the trade-off between semantic coherence and exclusivity to choose the optimal *K*. In particular, we locate the point in the graph in which exclusivity and semantic coherence plateau, such that an increase in the number of topics does not yield a big increase in exclusivity and small decrease in coherence. As shown in Fig. S1 exclusivity augments steeply until around 50 topics, when adding more topics slowly leads to smaller increases in exclusivity. We choose 90 topics, the point at which we find the plateau in the exclusivity graph. We report a robustness check in Additional file 1 S1 using a lower ($K=70$) and a higher ($K=110$) number of topics. Moreover, in Additional file 1 we fit an Structural Topic Model with a similar equation than (1), but with the interaction term $T_{t}Q_{i}$. We then repeat the ‘search *K*’ analysis and find similar results, providing further robustness to our choice of $K=90$.

We label the topics following the recommendations by Debortoli et al. [16]. Specifically, two members of the research team independently label each topic guided by the top 10 terms chosen using the frequency-exclusivity scoring ([FREX]; Roberts et al. [55]) after careful reading a sample of 25 documents associated with each topic selected using the *findThoughts* function of the *stm* package. A third member reads the designations resulting from this task and decides the final label. Disagreements are solved by discussion. We distinguish between three types of topics based on their inner consistency – clear topics (CL), multi-topics (MT), and boiler-plate topics (BT). Clear topics, as the name suggests, are readily interpretable topics. Multi-topics are topics that include more than one clearly interpretable topic. Boiler-plate topics tend to be centered around certain words but do not have an inner consistency, e.g., a topic consisting of posts that contain the word ‘look’. While boiler-plate topics provide little intrinsic meaning, they are helpful to encapsulate noise and avoid spillovers to other more meaningful topics (DiMaggio et al. [19]). For reference, we label boiler-plate topics with the word they seem to cluster around, but discard these topics from our analysis.

### Sentiment analysis

We study the sentiment present in the topics of the ‘r/jobs’ posts using the NRC emotion lexicon (Mohammad and Turney [44]), which identifies sentiment across polarity (positive and negative) and the eight basic emotions (fear, anger, sadness, disgust, joy, trust, surprise, and anticipation) according to the theory by Plutchik [52]. The NRC lexicon (Mohammad and Turney [44]) has shown better performance in word-emotion lexicons than other approaches (Kušen et al. [37]) (in Additional file 1 S4 we check the robustness of our results with respect to other methods). To characterise the sentiment across topics we select the top 1000 documents (∼2%) with highest prevalence for each topic and measuring the mean sentiment. This way, we are able to better understand topics beyond their thematic aspect. In Additional file 1 S4 we report an additional sentiment analysis for the full corpus of topics.

### Difference-in-differences event study

Equation (1) reveals how topics changed with the onset of the pandemic and addressed the first research question. However, this equation does not distinguish between quit and non-quit discourse, which is important to link our study to the causes of the Great Resignation. To study how the quit discourse changed relative to the non-quit work discourse we analyze the change in the topics defined by the Structural Topic Model outlined above using a difference-in-differences analysis model. One way of doing this would be to include an interaction term as in Eq. (4) (see Additional file 1) that signals the period before and after the onset of the pandemic. However, this specification would make the implicit assumption that the difference in topic prevalence between the control and the treatment group before the pandemic was constant over time. When this assumption is violated, the coefficients of Eq. (4) are misleading.^2^ To overcome these issues, we use a difference-in-differences equation akin to an event study, where we include a set of time dummies and interaction terms corresponding with the quarter in which the post was published. In particular, we estimate the following model,

2 $$ y_{it}=\alpha + \beta _{1}Q_{i}+\sum _{q=-7}^{7}\beta _{2}^{q}T_{t}^{q} + \sum_{q=-7}^{7}\beta _{3}^{q}Q_{i}T_{t}^{q} +\epsilon _{it}, $$

where $y_{it}$ is the topic prevalence in posts *i* published at time *t*, and $t={2018q1, \ldots, 2021q4}$ is measured quarterly. $Q_{i}$ is a dummy variable that takes a value of 1 if the post is quit-related (treatment group) and 0 if it isn’t (control group). $T_{t}^{q}$ is a set of dummies corresponding to 15 quarters, seven pre-pandemic, one tagging the start of the pandemic (Q1, 2020), and seven during the pandemic. Each takes a value of 1 for the specific quarter that they indicate, and zero otherwise. The very first quarter (Q1, 2018) is left out of the equation. The vector of coefficients $\beta _{2}^{q}$ is their corresponding coefficients. The coefficient $\beta _{1}$ shows the overall difference in topic prevalence between the treatment and the control group, while the vector of coefficients $\beta _{3}^{q}$ shows the differences between the groups over quarters (*q*). The equation also includes a constant *α* and an error term $\epsilon _{it}$. The topics in this difference-in-differences specification correspond to those identified by the main Structural Topic Model, described in the section above. In this way, we guarantee that we discuss one set of topics throughout the whole manuscript and address our two research questions.

### Multiple regression with quits as the outcome variable

Thus far we have laid out estimation strategies that help us understand whether the pandemic changed the relationship between quit- and nonquit- related discourse. Here we more directly ask whether certain issues (as identified by the topics) could have led to quit mentions and whether these relationships changed with the onset of the pandemic. Hence, we have quit mentions as our outcome variable, and select topics as the variables of interest. We think of these issues as either factors that increase the probability of quitting (push factors) or factors that reduce it. We estimate the following model using the logistic regression

3 $$ Q_{it} = \boldsymbol{X_{it}^{\prime } \beta}+ \boldsymbol{Y_{it}^{\prime } \gamma} + \boldsymbol{T^{\prime }_{t} \delta} + \varepsilon _{it}, $$

where $Q_{it}$ is a binary variable indicating whether a post is quit-related $(1)$ or not $(0)$, $\boldsymbol{X_{it}}$ is a set of topics indicating push factors, whose set of coefficients ***β*** we are interested in. $\boldsymbol{Y_{it}}$ is a set of topics related to factors that may reduce one’s intention to quit and ***γ*** the respective coefficients. $\boldsymbol{T_{t}}$ is a full set of time dummies, indicating months, which control for economy-wide time-variant factors that also affect the quit rate, such as the rate of job openings and other business cycle changes. These effects are captured in coefficients ***δ***.

We use a sample of posts with the same composition of quit- and nonquit- related posts as in the general ‘r/jobs’ population of posts. From the 78 interpetable topics we treat *mental health*, *hating job*, *work-related distress*, and health issues* as potential push factors. We choose *hating job* since it relates to toxic work culture and is similar to *hate my job & want to quit*, but is not confounded with posts being quit-related. In addition, we identify factors that should improve the conditions of a job and hence lessen the intention to quit: (flexibility of) *working from home*, *salary negotiations* and *job promotions*. We choose *working from home*, rather than *remote jobs*, since *working from home* is more related to the experience and *remote jobs* is more about users looking for remote jobs.

To test if the pandemic changed the relationship between the topics and quit mentions, we estimate the same model twice, once for the pre-pandemic period (January 2018–February 2020), and once for the period since the start of the COVID-19 pandemic (March 2020–December 2021). Significant differences in the estimated coefficients between the two models would suggest a change in the strength of the relationships, possibly as a result of the pandemic.

## Results

### The population of ‘r/jobs’

To contextualize our findings, we present our results on the demographic characteristics of the population of ‘r/jobs’. In the embeddings provided by Waller and Anderson [68] each subreddit has an age coordinate between −0.61 and 0.62, where a more negative value indicates a younger population relative to the general Reddit population; and a gender coordinate between −0.35 and 0.52, where a more negative value indicates a more male population relative to the general Reddit population. The ‘r/jobs’ age coordinate is 0.21 and gender coordinate 0.11. In other words, in comparison to the average subreddit, ‘r/jobs’ has an older population and a slightly higher female representation.

Next, we present the results from the sample of 400 users we manually examined to trace self-disclosed demographics. We were able to record self-disclosed gender for ca. 40%, age for ca. 29%, education level for ca. 38%, and country of residence for ca. 32% of all 400 users. We present the results in Table 1.

**Table 1:** Demographics of users of r/jobs. Reported are aggregates of self-disclosed characteristics found within the posting history of a random sample of 400 users (200 pre and 200 post the onset of the COVID-19 pandemic). Undergraduate and graduate degree statistics include both individuals reporting having completed as well as those reporting dropping out or currently studying a degree. Characteristics for ^*a*^ were identified on 158 (89 pre and 69 post 01.03.2020) authors, for ^*b*^ on 115 (68 pre and 47 post), for ^*c*^ on 153 (88 pre and 65 post), and for ^*d*^ on 127 (64 pre and 63 post)

	Before March 2020	After March 2020
Gender^*a*^		
Female	51%	68%
Age^*b*^		
17 years old or younger	10%	6%
between 18-34 years	78%	92%
between 35-49 years	12%	2%
Education^*c*^		
High school degree or less	14%	9%
Undergraduate degree	75%	76%
Graduate degree	11%	15%
Country of residence^*d*^		
U.S.	81%	70%
Canada	6%	8%
U.K	3%	13%
Elsewhere	10%	10%

These results complement those of Waller and Anderson [68] and, taken together, suggest that we are studying the Great Resignation through a population of mostly U.S.-based working age young adults. After the onset of the pandemic, the user population became more international, although the U.S. remained by far the most common country. Interestingly, after the onset of the pandemic, the female population became the majority. This finding may be caused by the disproportional burden women faced during the pandemic at work (Adams-Prassl et al. [1]), leading them to rely on community support to help them overcome such issues.

When interpreting these results, we must take into account that we are relying on self-disclosed information and that the probability that a user mentions their gender or country of residence may differ for different groups of users or might have changed over time. For example, during the pandemic users might have been more likely to report they were not from the U.S. to provide context to other redditors. Therefore, we provide the results in Table 1 to provide context, but we do not consider them reliable enough to incorporate them into our statistical analyses.

### Reddit and the U.S. Great Resignation

Considering that our sample of study is mostly U.S.-based and that a considerable amount of the Great Resignation media attention has been around this country, we focus our discussion within the context of the U.S. Note that we have the caveat that we cannot exclude users outside of the U.S. from our sample since it is infeasible to manually extract the geolocation of all users. Nonetheless, as we discuss in the following paragraphs, the evolution of the ‘r/jobs’ discourse resembles the dynamics of the U.S. labor market.

**Figure 1:**
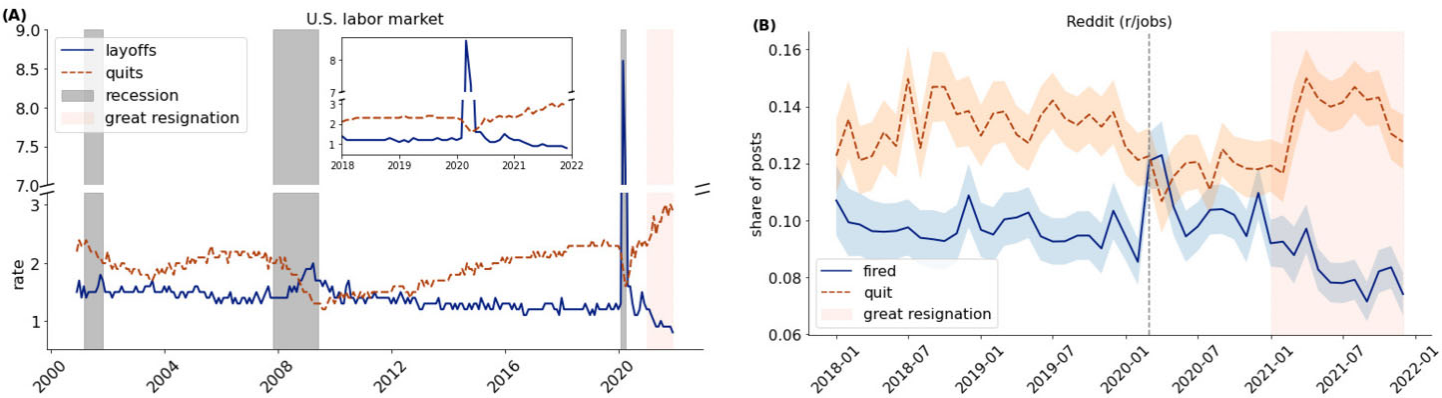
US labor market and ‘r/jobs’. (A) The U.S. quit and layoff rates from December 2000 to December 2021. Recession periods are marked with grey shaded areas. The top right corner zooms into the 2018-2020 time series for easier comparison with the Reddit time series. (B) The share of quit- and fire- related posts from 2018 to 2021. In both panels the dashed vertical line corresponds to March 2020, the orange shaded area to the Great Resignation period, and the frequency is at a monthly level

Figure 1A shows the U.S. quit and layoff rates from 2001 to 2021, portraying its pro-cyclical behaviour and the record high quit rates in 2021 (the top right corner zooms into the 2018-2020 time series). There is no clear start of the Great Resignation, but we know it is a 2021 phenomena, hence we highlight with an orange shaded area the year 2021 as the approximate period of the Great Resignation in the plots of this paper. As discussed in the introduction, the pro-cyclical behaviour of the quit rates contributed to the Great Resignation, but is unlikely to be the full story. In Additional file 1 S2 we argue this further by showing that the relationship between job openings and quits has weakened in the U.S. In said section, we also include a more detailed description and discussion of the U.S. labor market and the Great Resignation using administrative data.

To compare the dynamics of ‘r/jobs’ discourse and the U.S. labor market, we compare the evolution of the share of quit-related posts with the quit rate. We also label posts as fired-related using keywords and compare the share of fired-related posts with the layoff rate. As shown in Fig. 1, two years before the pandemic the shares of quit- and fired-related posts were roughly constant, similar to the rates in the U.S. labor market. In March and April 2020 the share of fired-related posts spiked and so did the layoff rate. In contrast, during this period, the share of quit-related posts and quit rate decreased. In 2021 the share of fired-related posts decreased constantly, while the share of quit-related posts increased rapidly and then remained roughly constant. These dynamics also match the fact that the quit rate increased in 2021 and the layoff rate decreased. For a quantitative comparison between the time series we calculate the correlation between the time series. The correlation between the Reddit posts and the U.S. economy quit time series is 0.57 (p-value $2.5\times 10^{-5}$), and the correlation with the layoff/fired time series is 0.60 (p-value $5.5\times 10^{-6}$). In other words, quantitatively the dynamics of ‘r/jobs’ are similar to the dynamics of the U.S. labor market.

In Additional file 1 S3 we present an additional analysis using posts’ flairs that shows that the ‘r/jobs’ discourse also qualitatively matches other aspects of the U.S. labor market such as an increase in job offers in 2021.

In this section we showed that the dynamics of the ‘r/jobs’ resemble those of the U.S. labor market before and during the pandemic and at the start of the Great Resignation. This finding provides validation to our study and suggests that by investigating the change in the text content of the ‘r/jobs’ posts we can better understand the Great Resignation. The next section provides additional validation by showing that with the start of the pandemic, topics related to working from home emerged and the discourse around commuting declined.

### Shifts in work discourse during the COVID-19 pandemic and Great Resignation

Here we address our first research question and study how the topics in ‘r/jobs’ posts changed since the start of the pandemic and relative to the pre-pandemic period. For example, were users more likely to talk about remote work, compensation or mental health after the onset of the pandemic? We describe and discuss the identified topics and then present the results for shifts in prevalence as well as the mean sentiment of each topic.

#### Identified topics

Out of the 90 topics of the Structural Topic Model, 68 of them are *clear topics*, 10 of them are *multi-topics* and the 12 remaining topics are noisy *boiler-plate* topics with difficult-to-identify themes (DiMaggio et al. [19]). In our analysis, we include only clear topics and multi-topics (78 topics in total). For brevity, we shorten multi-topics names to one or two theme and mark them with a star at the end of the name. Table S4 in Additional file 1 S5 reports the full name and type of all topics, as well as their most frequent and exclusive (FREX) terms. When interpreting changes in prevalence across posts generally, one must bear in mind that there are 90 topics, meaning that the expected average prevalence of topics is 1.1% (0.011 in the figures), such that changes might seem small in magnitude, but this arises from the large range of narratives discussed in r/jobs. When interpreting shifts in prevalence of multi-topics specifically, we nuance the findings since the changes could arise from changes in any topic or combination of topics.

From the 78 interpretable topics, we focus on those that are relevant within the context of the COVID-19 pandemic and the Great Resignation. We consider a topic to be relevant within this context if it is related to (i) remote work, given the major shift to remote work during the pandemic (del Rio-Chanona et al. [18]); (ii) health, anxiety and mental health, since this was often discussed in the media (Cook [14], Sull et al. [65]) as a cause of the Great Resignation; (iii) quitting or switching jobs, which is what the Great Resignation is about, (iv) job searching, since this may precede a quit, and (v) earning and careers, given that the predominant reason for people switching jobs is a higher salary (Hall and Lazear [25]). In the following paragraphs we explain the topics that fall within each category and what they are about.

The topics *working from home* and *remote jobs* include discussions about issues and anecdotes of remote working conditions, as well as searching for remote jobs. *Remote jobs* is mostly about searching for remote jobs, while *working from home* is more about narrating working from home experiences, jobs or related issues. In contrast to these topics, the topic *community, moving for a job* includes worries about taking a job that is far from home and discussions of whether it would be good to relocate to reduce commuting time.

Topics about health issues include *Work-related distress*, *mental health*, and the multi-topic *health issues/healthcare job/scheduling* (from here onward referred to as health issues* for brevity). *Work-related distress* and *mental health* are similar topics that are centered around negative psychological experiences and discuss anxiety, stress and depression – the three mental illnesses most discussed in workplace settings (Harvey et al. [27], Joyce et al. [32]). The former topic captures job-related distress and includes posts about feeling lost, stressed, anxious and/or overwhelmed at work. In the latter topic, posts discuss mental health concerns and explicit psychological disorders such as depression and anxiety. Health issues* includes worries about health, experiences by healthcare workers, and general work scheduling issues. These issues seem to be clustered together since discussing health issues can include requesting/scheduling appointments (*request* is included in the topic’s top 10 terms), which is also a work management issue. This term also pops-up when discussing healthcare jobs, as these are characterized by work shifts. As explained previously, we nuance findings when interpreting the results of this multi-topic due to identification issues.

Topics related to job quitting intentions and experiences, include *switching jobs, guilt about leaving for a better job, quit, resignation letters* and *quitting a new job*. Two topics stand out in the context of the Great Resignation. In *switching jobs* people discuss wanting to switch their job and question about how to handle a job switch. The other topic that stands out is *hate job & want to quit*, which includes narratives of employees fed up with their jobs, among others, due to toxic workplaces (one of the FREX terms). Toxic workplaces and job switching are two of the proposed drivers of the Great Resignation (Sull et al. [65], Krugman [36]). The topic *hating job* is not related to quitting, but related to toxic work, as people mention they hate or strongly dislike either their co-workers or the tasks they do in their job.

With respect to job searching, the most general related topic is *job searching*, where people report their experiences on searching for a job and ask for advice. There are also two other self-explanatory topics: *online job search* and *difficulty finding a job*. One last topic related to job searching is the multi-topic *looking for jobs / sales related questions* (from here onward referred to as looking for jobs* for brevity), which includes posts about looking for jobs mostly on sales and questions related to sales.

Finally, there are also topics related to earnings and careers, for example, *salary negotiations*, where people ask how to negotiate salary in a job offer or ask for a raise in their current job, *online jobs to make extra money*, which is about side jobs, mostly remote, to earn extra money, and *make money*, where users, many of them teenagers, ask about how they can obtain some earnings. Two other topics related to people’s careers are *job titles, promotions*, where users ask about changes in their job title and/or mention they got a job promotion; and *job offers issues*, which include posts about not hearing back after accepting a job offer, but also asking for advice about what to do when the user accepted one job offer, but then got a better one.

#### Shifts in the work discourse

To understand how the work discourse changed with the pandemic, we use the estimates obtained from the Structural Topic Model with the prevalence of each topic as an outcome ($y_{i}$ in Eq. (1)) across documents and time. As a first approach, we are interested in the average difference in the prevalence of topics before and after the onset of the pandemic. In later sections, we explore temporal and quit- nonquit -related variations in more detail.

**Figure 2:**
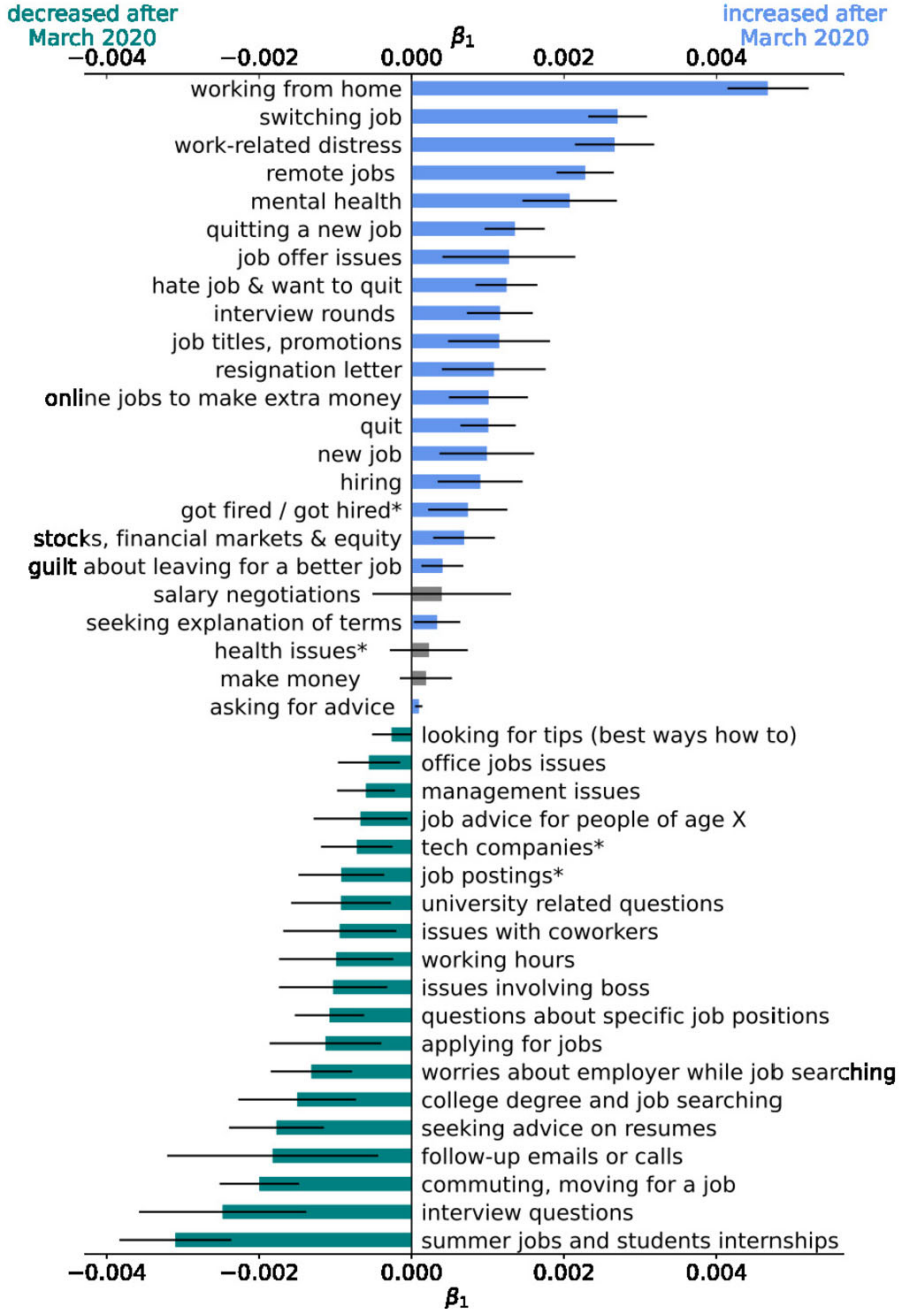
Shifts in overall topic prevalence. This figure shows the time coefficient of the regression in Eq. (1). Blue bars indicate a positive coefficient with a p-value below 0.05, while green bars indicate a negative coefficient with a p-value below 0.05. In grey, three relevant topics that do not present a significant change (p-value above 0.05) are added. Topics with a star at the end correspond to multi-topics, and the name may be shortened to a single topic. For the full name of multi-topics, please see Table S4

Figure 2 summarizes the changes in topic prevalence; it shows the coefficients $\beta _{1}$ from Eq. (1) where the p-values are significant (below 0.05). 39 out of 78 topics changed their average prevalence significantly after the onset of the pandemic. Topics that increased their prevalence are colored in blue, while those that decreased in green. There are three additional topics colored in grey that did not present a significant change in the average prevalence but are of research interest in the context of the Great Resignation. Namely, we added the topics *salary negotiations*, *make money*, and health issues*, as they are related to wages and health.

The topics with the largest increase in prevalence are: *working from home*, *switching jobs*, *work-related distress*, *remote jobs*, and *mental health*. Moreover, five topics related to job quitting increased their prevalence significantly – *quitting a new job*, *hate job & want to quit*, *resignation letter*, *quit*, and *guilt about leaving for a better job*. Among the topics that show the greatest decline in prevalence we have *commuting, moving for a job*. These changes resemble some known facts about how work changed due to the pandemic (e.g., the rise of remote work and decrease in commute (Brynjolfsson et al. [11])) and the Great Resignation (e.g., an increase in quit rates).

We perform a structural break Wald test to understand whether the pandemic caused the change in topics’ prevalence. This test determines whether the coefficients of the linear trend fitted to each topic time series before and after the pandemic differ. Therefore, this test allows us to discard changes due to a pre-existing upward or downward trend. We find that the five topics with the largest increase in prevalence show significant structural breaks in Q1 of 2020 (p-value of 0.05 or lower). Except for the *summer jobs and student internships* topic, the top four topics that decreased their prevalence the most also show a structural break in the Q1 of 2020. Other topics that declined in prevalence with a level break in Q1 of 2020 (significant at 0.05 or lower) are *applying for jobs* and *management issues*. These results suggest that it was indeed the pandemic that caused the changes in the prevalence of several of the topics discussed above that are related to the Great Resignation.

**Figure 3:**
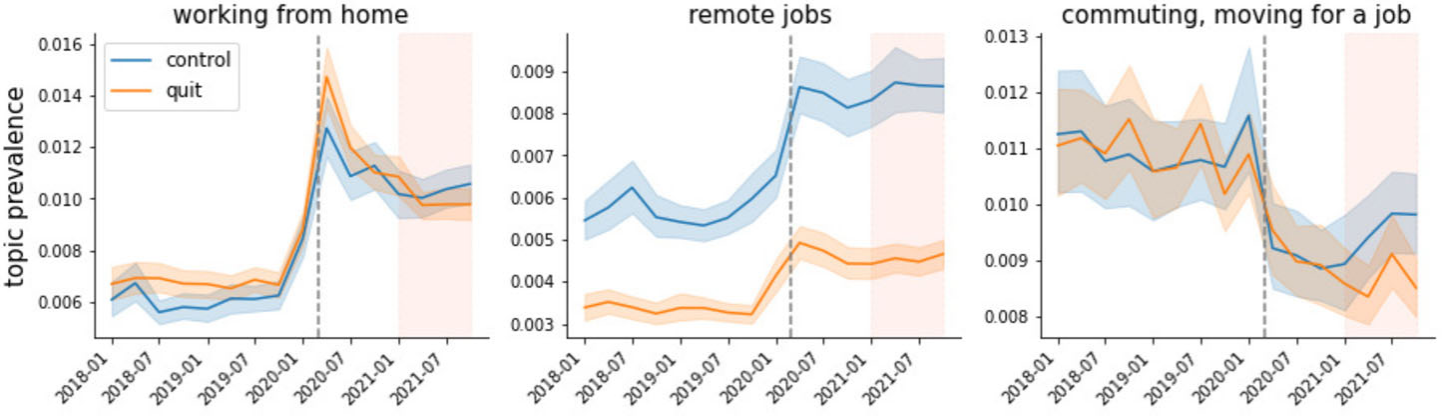
Dynamics of topics related to working conditions that changed during the pandemic. This plot shows the prevalence across time (in quarters) for the topics *working from home*, *remote jobs*, and *commuting*, *moving for a job*. Quit- and nonquit- related posts are in orange and blue, respectively. The shaded areas around the time series denote the 95% confidence intervals. The dashed grey line marks the onset of the pandemic (March 2020), while the shaded area represents the period of the Great Resignation (2021)

**Figure 4:**
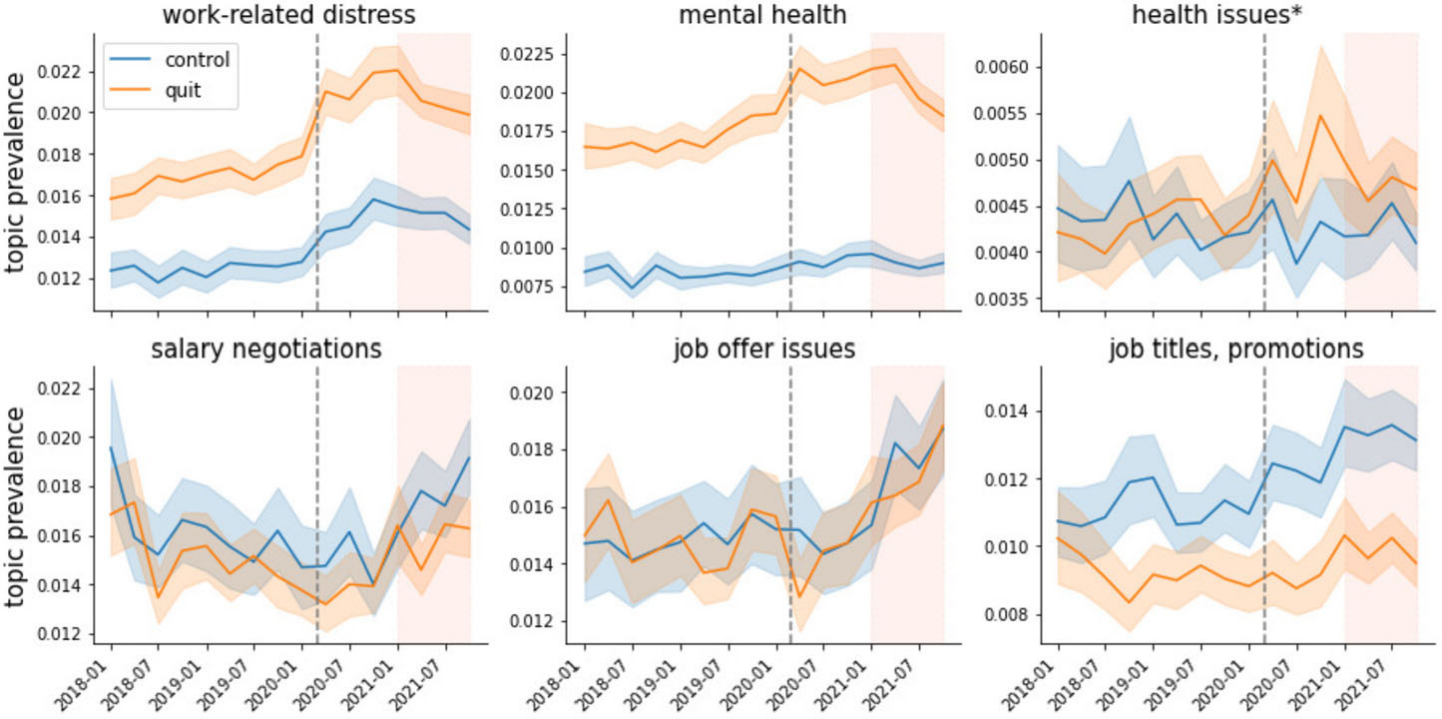
Dynamics of topics that reflect possible positive or negative effects of the pandemic and the Great Resignation. Prevalence across time (in quarters) for the topics *work-related distress*, *mental health*, and *health issues/jobs in healthcare/scheduling* issues on the top row and *salary negotiations*, *job offer issues*, and *job title, promotions* on the bottom row. Quit- and nonquit- related posts are in orange and blue, respectively. The shaded areas around the time series denote the 95% confidence intervals. The dashed grey line marks the onset of the pandemic (March 2020) while the shaded area represents the period of the Great Resignation (2021)

**Figure 5:**
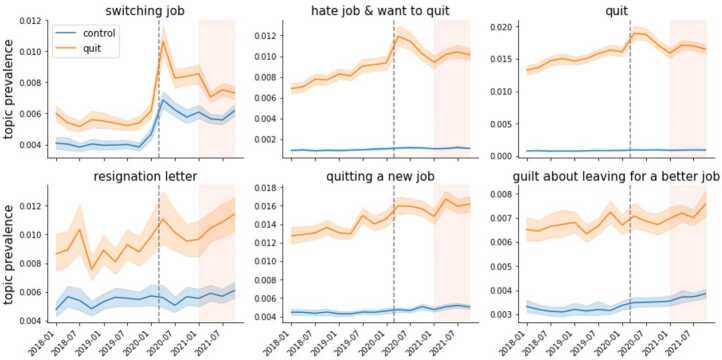
Dynamics of topics about quitting. Prevalence across time (in quarters) for topics *switching job*, *hate job & want to quit*, and *quit* on the top row and *resignation letter*, *quitting a new job*, and *guilt about leaving for a better job* on the bottom row. Quit- and nonquit- related posts are in orange and blue, respectively. The shaded areas around the time series denote the 95% confidence intervals. The dashed grey line marks the onset of the pandemic, while the shaded area represents the period of the Great Resignation (2021)

The labor market changed substantially between the first quarters of 2020 and 2021 – from record high fires to a record high quit rate – hence, it is also likely that the work discourse changed within this period. To analyze these finer-grained changes, we plot the quarterly dynamics of topics related to the pandemic and the Great Resignation. We split the topics across figures by putting together topics related to working conditions that changed during the pandemic (Fig. 3), topics that reflect possible positive or negative effects of the pandemic and the Great Resignation (Fig. 4), and topics about quitting (Fig. 5). For the dynamics of the rest of the topics please see Additional file 1 S5.

Figure 3 shows that after the onset of the pandemic, the topics *working from home* and *remote jobs* increased their prevalence dramatically and that the prevalence of *commuting, moving for a job* decreased sharply. While the prevalence of *remote jobs* and *commuting, moving for a job* remained roughly constant between 2020 and 2021, *working from home* peaked immediately after the onset of the pandemic but decreased its prevalence shortly after. This latter pattern suggests that there was a period of sense-making, during which individuals were unsure about how to deal with the unexpected shift to the new work scheme. The decline in discussions around commuting and moving for a job mirrors the increase in discussions around remote work and working from home. The pandemic-related trends around remote work, home office, and commuting confirm what we already know from other studies (Brynjolfsson et al. [11], McFarland et al. [43]), landing additional validation for using Reddit as a source of data to monitor the development of job-related attitudes.

(How) did the Great Resignation change the work discourse? Fig. 4 shows that the prevalence of topics related to job opportunities increased during the Great Resignation; in contrast the prevalence of topics related to detrimental effects decreased. *Salary negotiations*, *job offer issues*, and *job titles, promotions* increased their prevalence in 2021, resembling the improving labor market conditions for workers during the economic expansion. This point also comes across in Fig. S13 in Additional file 1 (fourth row), which shows that *difficulty finding a job* decreased its prevalence in 2021. In the second half of 2021 the topics *work-related distress*, *mental health*, and health issues* also decreased their prevalence, particularly among quit-related posts (we will examine this latter point more closely in the next section). The dynamics of the topics discussed here reflect how the labor market conditions improved in 2021 and suggest that these improvements may have given some relief to the detrimental effects of the pandemic.

**Figure 6:**
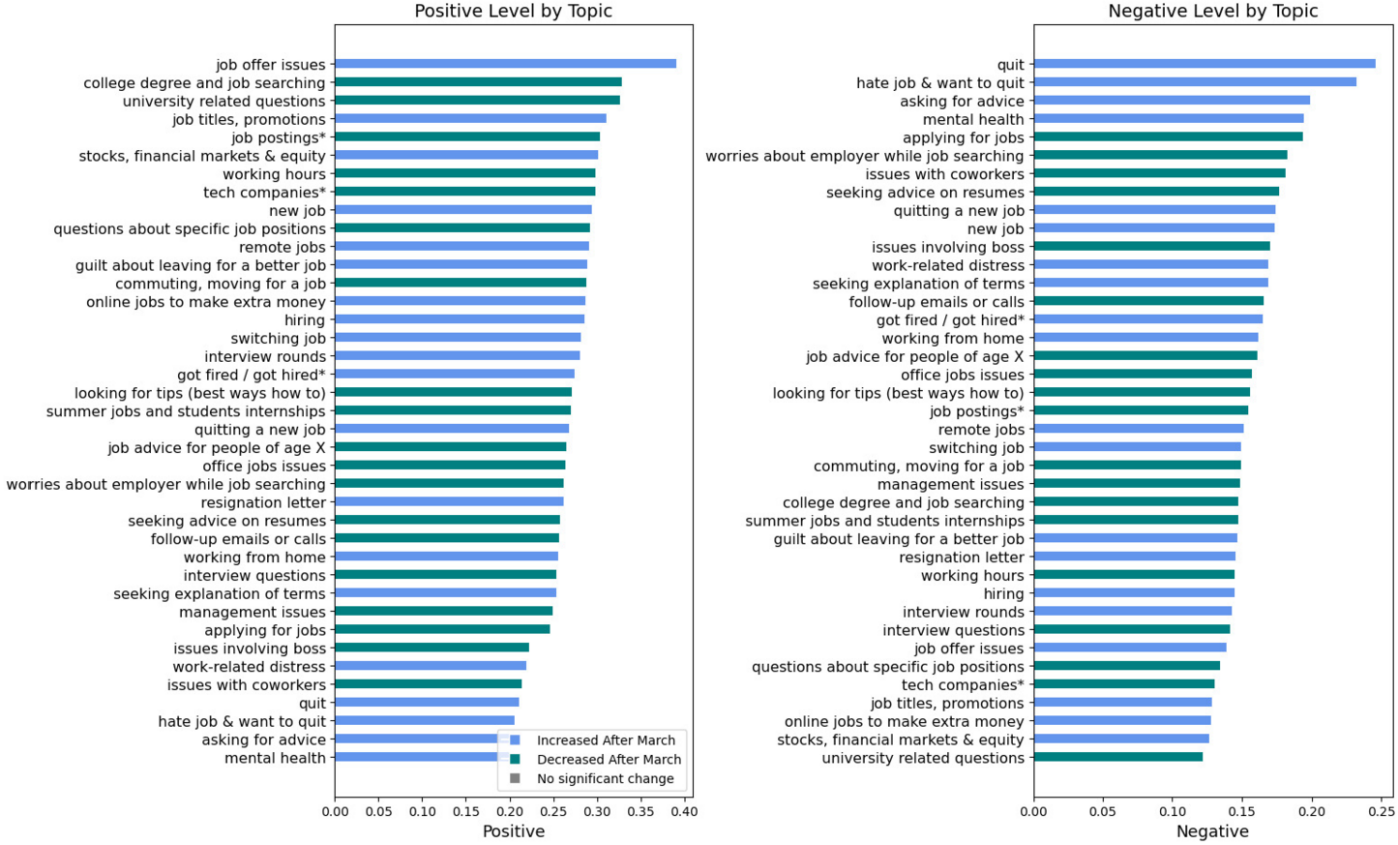
Topic Sentiment. This figure shows the average positive (left) and negative (right) sentiment of the topics that significantly changed their prevalence (see Fig. 2). Those colored in blue increased their prevalence, while those in green decreased it

The dynamics of posts related to quitting present a similar narrative. *Switching jobs*, one of the topics that increased the prevalence the most, presents a sharp increase at the onset of the pandemic; afterward, the prevalence decreased but remained above pre-pandemic levels (see Fig. 5). This finding supports what some economists have argued – the Great Resignation is more of a Great Reshuffling with workers switching jobs rather than leaving the labor force (Krugman [36]). The prevalence of *hate job & want to quit* and *quit*, the two most negative topics about quitting (see Fig. 6), increased sharply in the Q1 of 2020 – this was a significant structural break with a Wald test with respective p-value below 0.05 – and then decreased considerably by the start of 2021. In contrast, less negative topics about quitting, such as *resignation letter*, and topics related to quitting for a new job, such as *quitting a new job* and *guilt about leaving for a better job*, increase their prevalence during 2021. Taken together, the changes in prevalence of topics presented in Figs. 4 and 5 suggest a change in mood around quitting during the Great Resignation – from quitting out of despair in the pandemic to quitting for a better job opportunity.

The findings suggest that, while quits were on the rise long before the pandemic, the pandemic intensified people’s intentions to quit. The topics around quitting changed both at the onset of the pandemic, and during the Great Resignation. The start of the pandemic induced concerns about mental health, and work-related distress. However, some of these concerns were resolved when the Great Resignation started, likely due to better employment opportunities. People talked less about quitting because they hated their job, and more about negotiating salaries and discussing job offer issues. Some of the people quitting in 2021 might have strongly disliked their job in 2020, but did not quit until 2021, when there were better employment prospects. Others, likely the ones that felt guilty about quitting, might not have disliked their job but left it for a better employment opportunity.

#### Sentiment analysis within topics that changed their prevalence

To further understand how the mood around work discussions changed after the pandemic, we report the positive and negative sentiment of the topics that significantly changed their prevalence after the pandemic. Figure 6 shows the mean positive and negative sentiment of the topics; those increasing in prevalence post-pandemic are colored in blue, while those decreasing are shown in green. The 5 most negative and positive topics include both topics that increased their prevalence (blue) and decreased their prevalence (green) after the pandemic. Among the top 5 negative topics are *hate job & want to quit*, and *mental health*, that increased prevalence but also *applying for jobs* that decreased. The top 5 positive topics are mostly related to job searching and included *job offer issues* and *college degree and job searching*, which increased and decreased prevalence respectively.

The intermixed colors in Fig. 6 suggest no correlation between sentiment and change in prevalence. We confirm this lack of correlation by measuring the point biserial correlation between mean sentiment of topics and a dummy variable denoting whether the topic increased or decreased prevalence. In Additional file 1 S4 we analyse other sentiments and also find no correlation between any of the sentiments (see Table S3). We also report the evolution of sentiment scores across the full sample of ‘r/jobs’ post, these time series show no significant change after the pandemic. This finding suggests that there is no clear, enduring change in trend of sentiments following the pandemic for the whole population. In other words, the pandemic impact varied across individuals; while some encountered work-related distress—typically linked with negative sentiment—others benefited from an uptick in job offers and promotions, leading to positive sentiments. Thus, overall, sentiment levels at the aggregate r/jobs population level remained relatively stable.

### Quit-related discourse changes and the causes of the Great Resignation

Having documented how the general work-related discourse changed in ‘r/jobs’ after the onset of the pandemic, we turn our attention to specific changes in the quit-related discourse. To understand how the quit-related discourse changed not only relative to the pre-pandemic levels, but also relative to posts that are not quit-related (i.e., the control group), we estimate an event study style difference-in-differences model as outlined in equation (2).

We present the results of the difference-in-differences analysis for all the 78 topics in Table S5. Here in the main text, we focus on the topics for which the difference-in-differences analysis satisfies the parallel trends assumption (or that have at most a pre-trend of one quarter) and that are relevant in the context of the Great Resignation.

*Mental health*, *work-related distress* and health issues* significantly increased their prevalence among quit-related posts relative to nonquit-related posts since the start of the pandemic. *Work-related distress* increased the prevalence by 11.4 percent among nonquit-related posts since the pandemic, and by 17.5 percent among the quit-related ones. The average relative difference is 6 percentage points (pp) for the quit-related posts. *Mental health* increased by 5.5 percent among the nonquit-related posts since the pandemic, and by 15.6 percent among the quit-related ones, a 10 pp difference.

This finding portrays once again the strong distress that people who wanted to quit their job in 2020 were in. Towards the end of 2020 the relative difference in the prevalence of *mental health* and *work-related distress* decreased, likely reflecting the improved labor market conditions. Nonetheless, it remained higher than in the pre-pandemic period. We take this as suggestive evidence that, in contrast with the pre-pandemic years, since the pandemic, including the period covering the first year of the Great Resignation, people that are quitting are more concerned with mental health and emotional stress.

The topic health issues* follows a similar pattern as *mental health* and *work-related distress*, but the relative change is smaller in magnitude. Its prevalence increased by 8.3 percent among the nonquit-related posts, and by 13.5 percent among the quit-related ones, an average difference-in-differences of over 5 pp. As discussed in the previous section health issues* is a multi-topic. It includes posts related to patients issues such as scheduling and healthcare workers treating patients, among other things. Therefore we cannot clearly conclude what part of health issues drives the pattern, and we are unable to further disentangle the topics by, e.g., analysing the most common word-counts (see Additional file 1 S5). At most we show evidence that health issues were predominant in the quit-related discourse in the period of the pandemic.

**Figure 7:**
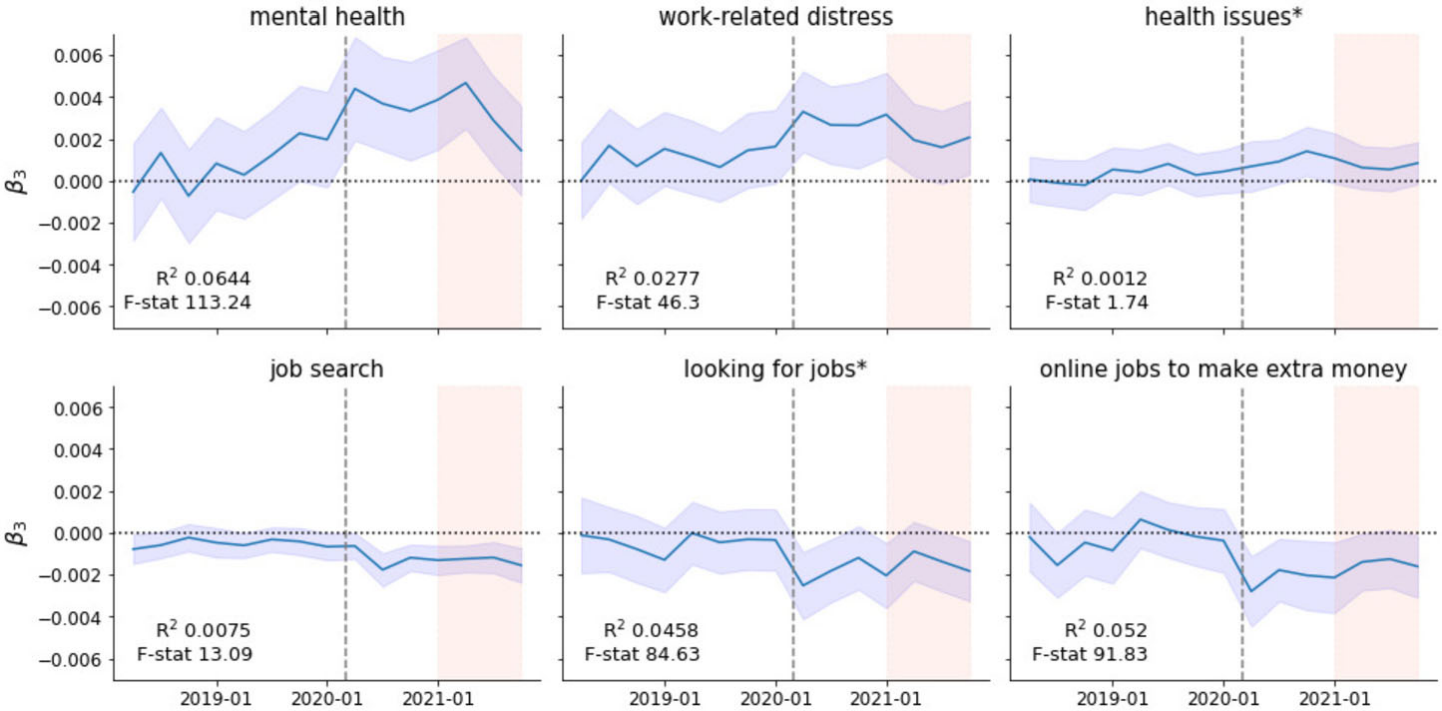
Difference-in-Differences analysis for selected topics. Relative changes in prevalence in selected topics among quit-related posts. Positive values indicate an increase in prevalence and negative values a decrease. The dashed grey line marks the onset of the pandemic (March 2020), while the shaded area represents the period of the Great Resignation (2021). The frequency is quarterly

The difference-in-differences analysis reveals that people seem to be less worried about finding a new job when talking about quitting after the onset of the pandemic. As the bottom panels of Fig. 7 show, topics related to job searching, such as looking for jobs*, *online jobs to make extra money*, and *job searching* decreased their prevalence in quit-related posts in comparison to the control group. Perhaps surprisingly, topics that portrayed a more positive status of the labor market (i.e., *salary negotiations*, *job titles, promotions*, and *job offer issues*) did not show a significant difference between the treatment and the control group (see Table S5). This does not mean that these issues are not relevant for workers quitting their jobs, but that they changed equally for both groups.

**Figure 8:**
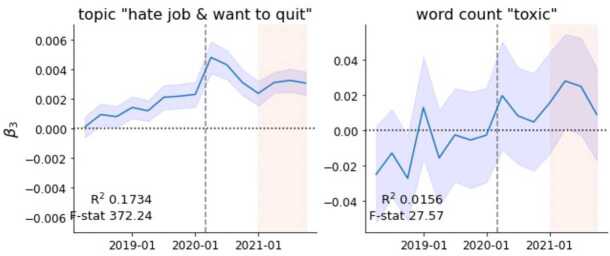
Difference-in-differences analyses. Relative changes in prevalence for the topic *hate job & want to quit* among quit-related posts. Additionally, the word count of the term “toxic” is presented. The dashed grey line marks the onset of the pandemic (March 2020), while the shaded area represents the period of the Great Resignation (2021). The frequency is quarterly

Another topic worth discussing in the context of work mental health and toxic work environments is *hate job & want to quit*. While this topic increased its prevalence among quit-related posts relative to the control group after March 2020, we cannot conclude the pandemic caused this increase since this topic has an upwards pre-trend. As shown in Fig. 8 and in Table S5, the prevalence of *hate job & want to quit* grew faster among quit-related posts pre-pandemic than in the general work discourse. This pre-pandemic increase was partly due to the word ‘toxic’ being used more often (see Fig. 8). Nonetheless, it still holds that in the first three quarters of the pandemic *hate job & want to quit* increased its relative difference in prevalence more sharply than before the pandemic. This difference then converged back to the pre-pandemic trend in 2021. These results suggest that, although the pandemic was not a root cause of increasing quits due to toxic work environments, it may have exacerbated this problem in 2020.

Taken together our results show that, among people that were talking about quitting, mental health concerns increased after the onset of the pandemic and before the Great Resignation. This finding suggests that distressing experiences at work and concerns about work-related mental health may have increased people’s motivation to quit. When more vacancies opened in 2021, some workers may have taken the opportunity to quit and switch jobs, as the increase in the prevalence of *switching jobs* would suggest, or leave the labor force. The Great Resignation also seems to have brought some relief to mental health concerns, particularly towards the end of 2021, where we see mental health-related topics decrease their prevalence towards pre-pandemic levels.

### The relationship between mental health concerns and quitting: multiple regression results

Here we present the results of estimating equation (3). For the estimation we use a logit regression because the outcome variable is binary (i.e., whether a post is quit-related or not). Model (1) of Table 2 shows the results for the pre-pandemic sample, and model (2) for the sample of the pandemic period. We aim to answer the following two questions: (a) Which topics remain associated with quit-mentions *after* controlling for other topics that are possible confounders? and (b) has the strength of the relationship between the topics of interest and quit-mentions changed since the onset of the pandemic? On the side of push factors, we find that the associations between *mental health, hating job,* and *work-related distress* on the one hand, and quit-mentions on the other are all significant, and their magnitude did not change since the start of the pandemic. The pandemic increased the incidence of these push factors (extensive margin), but it did not intensify the extent to which such incidence leads to quits (intensive margin). Hence, the pandemic contributed to the Great Resignation through the extensive margin – by creating greater mental distress in the workplace. In particular, *mental health* proves the best predictor of quit-mentions. A post about mental health (prevalence of one) is almost twice as likely to be quit-related than a post which is not about mental health (prevalence of zero), keeping other topics constant. However, topics rarely have prevalence close to 1 in a post, so another way to express the result would be to ask how the probability of quit-related posts increases if we move from the 5th to the 95th percentile of mental-health-related posts. Such move corresponds with an increase in the quit probability of 6.1 percent. A similar move in *hating one’s job* is associated with an increase in the probability of a quit-related post of 5 percent. And in the case of *work-related distress*, we find 1.7 percent higher quit probability.^3^

**Table 2:** Explaining Quit Considerations. Results from a logit regression specified in equation (3). The first model includes the months since January 2018 and until the end of the pre-pandemic period (February 2018), The second model includes the months since the start of the COVID-19 pandemic, and until the end of our period of observation (March 2020–December 2021)

	Model (1)	Model (2)
	Jan 2018-Feb 2020	Mar 2020-Dec 2021
Mental health	1.968***	1.919***
	(0.179)	(0.175)
Hating job	1.262***	1.319***
	(0.147)	(0.128)
Work-related distress	0.342**	0.404***
	(0.149)	(0.110)
Health issues	−0.110	0.167
	(0.307)	(0.202)
Working from home	−0.461*	−0.461***
	(0.270)	(0.168)
Salary negotiations	0.121*	0.208***
	(0.0643)	(0.0644)
Promotions	−0.0279	−0.414**
	(0.155)	(0.167)
Log-likelihood constant only model	−5306.67	−5275.789
Log-likelihood	−5046.806	−4926.843
Pseudo R-squared	0.049	0.066
Observations	14,549	14,722

Robust standard errors in parentheses.

Significant at: *** p < 0.01, ** p < 0.05, * p < 0.1.

On the side of factors that reduce our incentives to quit, we find that *working from home* and *promotions* are indeed associated with lower probability of quit mentions. Additionally, in the case of *promotions*, we only find a significant positive relationship during the pandemic period, suggesting the possibility that the relationship between *promotions* and quit mentions intensified. Lastly, the coefficient of the topic *salary negotiations* is also significant and stronger in the pandemic period, but its positive sign is unexpected. One likely explanation is that they mention quits in conjunction with having received better salary in the new job. In terms of magnitude, *working from home* and *promotions* during the pandemic have a similar effect as *work-related distress*.

## Discussion

The COVID-19 pandemic shook the global labor market like no other economic recession that we have on record. It led to the Great Resignation in 2021, a record high quit rate in the U.S. and considerably high quit rates in other countries. While traditional economic forces, such as labor shortages and the resulting wage increases played an important role, the media is now often citing burnout, toxic work environment and desperation as leading causes. Such motivations cannot be easily studied using official survey and administrative data, and one remains sceptical of bombastic media headlines that use private sources of data or small-scale surveys. In this paper we take a different approach and use data from Reddit. We focus on ‘r/jobs’, where more than a hundred thousand people shared their work-related questions and concerns, discussing issues that emerge more organically and are more intimate than what official statistics can cover. Through studying the evolution of the work discourse using sentiment and text analysis, we shed light on how the pandemic affected workers and find some evidence that, along with the usual causes of quitting in recovery periods such as job switching, mental health concerns may have been one of the drivers of the Great Resignation. Here we summarize our main findings and discuss their implications.

Our first finding, which also serves as validation, is that the evolution of the ‘r/jobs’ discourse resembles the dynamics of U.S. labor market. We show this first by comparing the shares of quit- and fired- related posts with the U.S. quit and layoff rates, and then by showing that since the pandemic topics related to remote work sharply increased their prevalence, while the topic about commuting declined. These findings contribute to the literature using social media data to study socio-economic systems (Nicolas et al. [46], Antenucci et al. [4]) and in some cases predict socio-economic changes (Bollen et al. [10], Lopez et al. [41]). Our study supports the use of Reddit as a real-time socio-economic observatory where one can study work and labor when administrative and official survey data is not available or to complement it. This emphasis also underscores the need for data access from online platforms, a necessity recently challenged by restrictions on APIs for platforms like Twitter^4^ and Reddit,^5^ and the decrease in online forums’ activity due to Large Language Models (del Rio-Chanona et al. [17]).

Using topic modelling, we document how the overall work discourse changed since the pandemic started. The topics that increased their prevalence the most include switching jobs, mental health, work-related distress, and remote work topics. In contrast, commuting is one of the topics that decreased their prevalence the most. Besides serving as validation, these results contribute to a more complex understanding of workers. We identify factors other than wages, such as mental health concerns, that may influence workers decisions. In this way, our work contributes to a growing literature studying meaning of work (Rosso et al. [60], Nikolova and Cnossen [47]), and can be a starting point to help guide future work that goes beyond modelling workers as wage-leisure maximizers and portrays human actions in a more realistic manner.

Our most important finding is that mental health and work-related distress likely contributed to the Great Resignation. Two results support this conclusion: First, mental health and work-related distress significantly increased their prevalence since the start of the pandemic, in quit-related posts relative to nonquit-related posts. Second, our multiple regression model revealed that mental health concerns and work-related distress topics are related to a higher probability of a post being quit-related, keeping other topics constant. We also find some evidence that some relief came to mental health concerns and work distress in the last two quarters of the Great Resignation, when the prevalence of *mental health* and *work-related distress* decreased. One possible reason for this relief may be the better labor market conditions of the year 2021. These findings contribute to the understanding of the causes of the Great Resignation. Furthermore, our work also contributes to the management literature by providing empirical evidence to the theoretical notion that shocks at the societal level can prompt turnover cognitions (Morgeson et al. [45]).

The last finding is that the relationship between mental health concerns and quitting did not change with the pandemic. In other words, people that suffered from mental health issues were not more likely to quit after the pandemic. Instead, it was the increase in prevalence of mental health concerns that contributed to the rise in quits. This result contributes to the literature documenting that the pandemic had a considerable effect on people’s mental health (Xiong et al. [70]).

Our research is not without its limitations. First, our results are based on the user population of ‘r/jobs’, which is not representative of neither the world nor the U.S. labor force. Furthermore, it is difficult to extract the demographic characteristics and employment status of the users. As a result, we are not able to check if the treatment and control groups are affected by attrition. This is also a limitation since the pandemic had heterogeneous effects among people of different gender, age, and occupation (Adams-Prassl et al. [1], Cook [14], del Rio-Chanona et al. [18]). Second, we cannot guarantee that the quit-related posts translate into an actual quit – the fact that a user talks about quitting does not guarantee the person will indeed quit their job. Furthermore, if there is a quit, we do not know the precise timing. People may discuss a past quitting experience or planning to quit. Third, our results rely on sentiment analysis and topic modelling, methods that have limitations (Iliev et al. [31]). For example, topic modelling uses a bag-of-words approach that in our study leads to multi-topics, where different topics get bundled together due to the same words being used in different contexts.

Nonetheless, we have made strong efforts to reduce and understand the effects of these limitations. We leveraged data provided by Waller and Anderson [68] study to uncover the characteristics of ‘r/jobs’ users. Furthermore, we are one of the few studies (von Hippel and Cann [67], Seraj et al. [64]) using Reddit that go through several hundred of posts, follow authors posting history, and manually encode self-disclosed information to understand the composition of the sample under study. Our results show that our sample has more women and young workers than the U.S. working population. Given the strong challenges working women faced during the pandemic (Adams-Prassl et al. [1]) and studies suggesting that the Great Resignation was driven by young adults (Cook [14]), we consider our sample population to be of interest for the topic. Although we cannot guarantee that people who talk about quitting will actually quit, the most recent meta-analysis showed that, out of 57 variables, cognitions about quitting is the strongest predictor of quit behavior (Rubenstein et al. [61]). Furthermore, research on online social networks has shown that diagnoses of clinical depression can be predicted by verbalization of the experience in Facebook 6 months before the diagnosis is made (Eichstaedt et al. [21]). Finally, to overcome topic modelling limitations, we used a Structural Topic Model, the state-of-the-art in social science studies (Hannigan et al. [26]). We consider that, despite the mentioned limitations, the advantages of using Reddit data and Natural Language Processing methodologies outweigh the caveats. Digital trace data is available in real time and allows for more personal and in-depth expression than surveys. In this sense our work does not substitute but complements traditional studies based on surveys or economic models.

Finally, this work can help guide some policy and business strategies. Our results suggest that some of the distress caused by the pandemic (Gruber et al. [23]) are linked to working conditions. This underscores the importance of designing work policies to reach the 2030 mental health targets of the World Health Organization (World Health Organization [69]). Furthermore, our work suggests that businesses trying to retain their workers or hire new people should consider prioritizing the mental health of their workers through, for example, the (re-)design of jobs (Harvey et al. [27]) or by providing company-sponsored therapy sessions for employees (Joyce et al. [32]).

### Supplementary Information

Below is the link to the electronic supplementary material.

(PDF 4.9 MB)

## Data Availability

All data needed to reproduce code and results will be made available upon publication at the following zenodo repository 10.5281/zenodo.7619909.

## Footnotes

1 We fortified the keywords to match any number of whitespace between the words. In the text, * represents any number of characters allowing for different forms of words, e.g., *resign** captures *resignation, resigned, resign* and some typos, e.g., *resigning*. For some keywords, we specify which letters are allowed to follow a keyword, e.g., *fir[e,i]** matches *fired* and *firing* but not *firm*. Lastly, we allow for optional words in between keywords (*), e.g., *chang* (*) job* matches both *change a job* and *change job*.

2 To understand the problem, think of a hypothetical example where among quit-related posts mental health is trending upwards before the pandemic while it is constant among nonquit-related posts, but such that the average of the constant and the average of the trend are about the same. Since the start of the pandemic, the same trend among the quit-related posts continues, while the other group sees no change in the prevalence of mental health. In such setup the average post-pandemic difference in the prevalence of mental health between the groups will be significant, and we’ll conclude that it was the pandemic that causes the differences. In fact, it was just a continuation of a pre-pandemic trend in the treatment group.

3 The relationship between *health issues** and quits is not significant. This does not mean that *health issues* (that are not about mental health) did not play a role in explaining quits, but that we are not able to detect this effect. Most likely, this is because, *health issues* is a multi-topic that captures both personal health issues, issues raised by health sector personnel, as well as issues around scheduling general appointments. In other words, we do no have a homogeneous topic that can capture the effect of health issues.

4 https://www.forbes.com/sites/jenaebarnes/2023/02/03/twitter-ends-its-free-api-heres-who-will-be-affected/.

5 https://www.reddit.com/r/modnews/comments/134tjpe/reddit_data_api_update_changes_to_pushshift_access/.

## Abbreviations

STM: Structural Topic Modelling
