# Supplementary material for: Mental health concerns precede quits: shifts in the work discourse during the Covid-19 pandemic and great resignation
Source: EPJ Data Sci. 2023 Oct 12;12(1):49. doi: 10.1140/epjds/s13688-023-00417-2 (PMC10570174; doi:10.1140/epjds/s13688-023-00417-2)
Supplement: Supplementary file 1 — (PDF 4.9 MB) [file 13688_2023_417_MOESM1_ESM.pdf]

Supplementary Material for Mental Health Concerns Precede Quits: Shifts in the Work Discourse during the Covid-19 Pandemic and Great Resignation

R. Maria del Rio-Chanona[1,2†], Alejandro Hermida-Carrillo[3,†],
Melody Sepahpour-Fard[4,5], Luning Sun[6], Renata Topinkova[7], and Ljubica Nedelkoska[1,2]

[1] Complexity Science Hub, Vienna
[2] Center for International Development at the Harvard Kennedy School
[3] LMU Munich School of Management
[4] Science Foundation Ireland Centre for Research Training in Foundations of Data Science
[5] Department of Mathematics and Statistics (MACSI), University of Limerick
[6] The Psychometrics Centre, University of Cambridge
[7] Department of Sociology, LMU Munich
[†] These authors contributed equally
delrio.chanona@csh.ac.at, A.Hermida@lmu.de, Melody.SepahpourFard@ul.ie, ls523@cam.ac.uk ,
renata.topinkova@lmu.de, nedelkoska@csh.ac.at

S 1 Topic modelling robustness tests

In this section we present the search K analysis and robustness checks in more detail.

S 1.1 Search K

As discussed in the main text, we fit the STM varying the number of topics K in increments of 5. Figure S 1 shows the exclusivity, semantic coherence, held out likelihood and residuals for each value of K. We choose $K = 90$ since it is the middle point of the shaded area, where exclusivity plateaus and coherence is not too low.

To verify the robustness of the choice of number of topics we fit an STM similar to the one in the main text (specified by Eq. 1), but including interaction between quit- and nonquit- related posts. Due to computational constraints we cannot fit a full STM using Eq.2. Therefore we fit an STM with the following equation (specified by Eq. 1, but with an additional interaction term T_tQ_i.

$$y_i = \alpha + \beta_1 T_t + \beta_2 Q_i + \beta_3 T_t Q_i + \epsilon_i. \tag{4}$$

Here, the prevalence of a topic y in a post i is determined by T_t, a dummy variable capturing if the post was published before or since the onset of the pandemic (March 1st, 2020), Q_i, the dummy variable for quit- (vs. nonquit-) related posts, the interaction of both variables T_tQ_i, the constant term α, and the error term ϵ_i. We use Eq.4 since it is a reasonable compromise between Eqs. 1 and 2 for repeating the search K exercise. Figure S 2 presents the result for the STM fitted using Eq.4, showing almost identical results and validating our choice for $K = 90$. This is not surprising given that our sample of posts is 50% quit related and 50% nonquit.

S 1.2 Robustness to variations on the number of topics

To further verify the robustness of our main result, i.e., the shifts on the mental health discourse, we repeat the topic modelling analysis varying the number of topics K. We use one lower bound and one

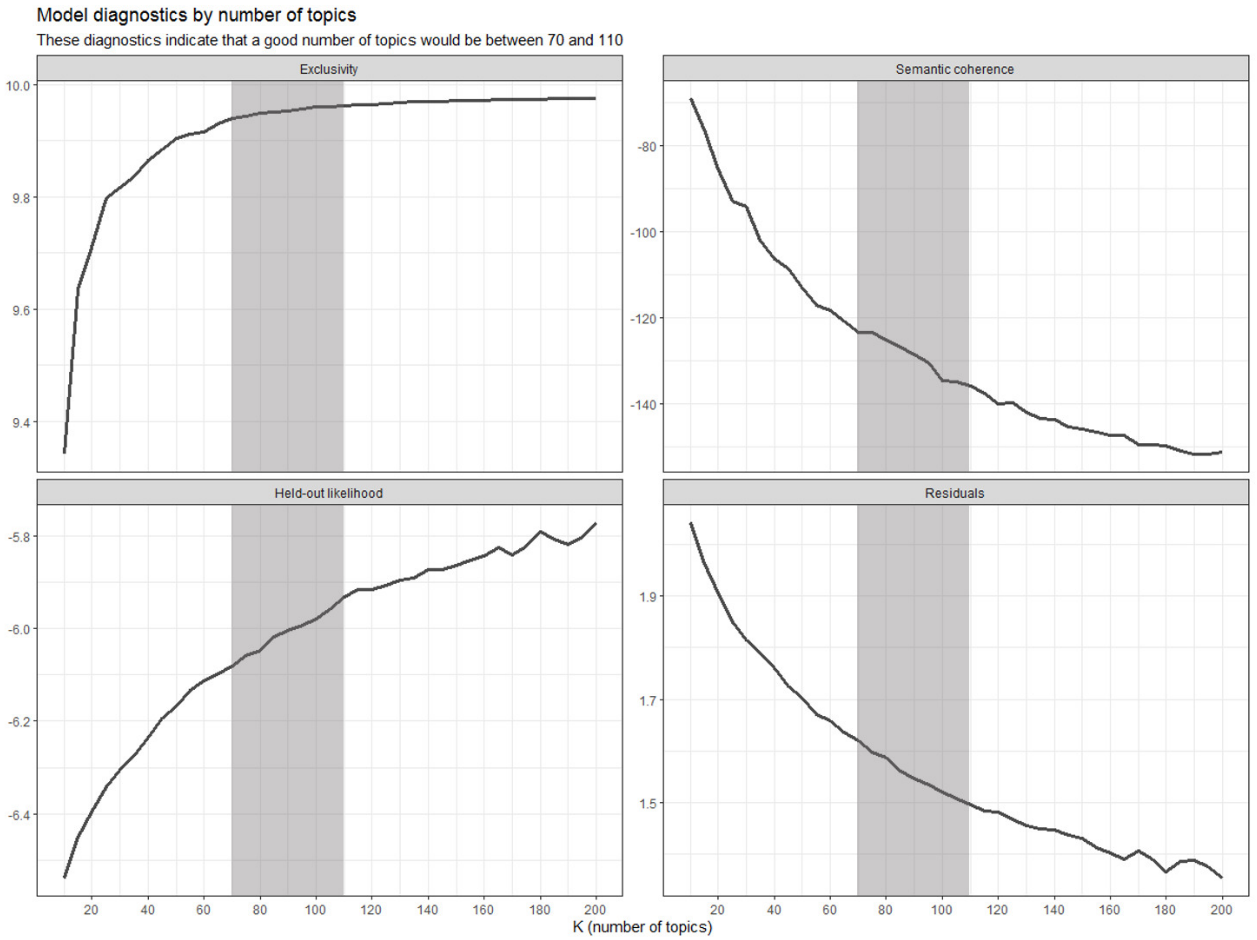

Figure S 1: Diagnostics of the STM as specified by Eq. 1 varying K from 5 to 200 in increments of 5. The shaded area represents the values of K we consider for the fit in the main text.

upper bound $K = 70$ and $K = 110$ respectively. While both solutions deliver a different set of topics (less and more exclusive respectively), we observe that our main results are mirrored in them. In the solution at $K = 70$, the term *mental health* is included in Topic 27, where it is interwined with the terms idiosyncratic to the topic *hating job* in our solution at $K = 90$ (FREX: *hate, anxiety, miserable, stress, suck, absolutely, depression, tired, hate job,* and *mental health.* This topic shows both an increase across time in a model estimating eq. (1) ($\beta_1 = .002$, $p < .000$), as well as a significant interaction effect in a model estimating eq. (4) ($\beta_3 = .003$, $p < .000$). In the solution with $K = 110$, *mental health* is included in Topic 80 (the same position as in our solution at $K = 90$) with almost fully overlapping Top 10 FREX keywords. The results are similar in both the simple ($\beta_1 = .002$, $p < .000$) and moderated ($\beta_3 = .003$, $p < .000$) models. The topic *work-related distress* is found in the same position (Topic 1) at $K = 70$ and $K = 90$ and as Topic 108 at $K = 110$, and is represented by mostly identical Top 10 FREX keywords in both models. Computing overall time changes with eq. (1) and changes idiosyncratic to quit posts with eq. (4) shows positive and statistically significant increases in both models. Other relevant topics (e.g., *hate job & want to quit*) are also found in the models at $K = 70$ and $K = 110$ and show qualitatively identical results across time.

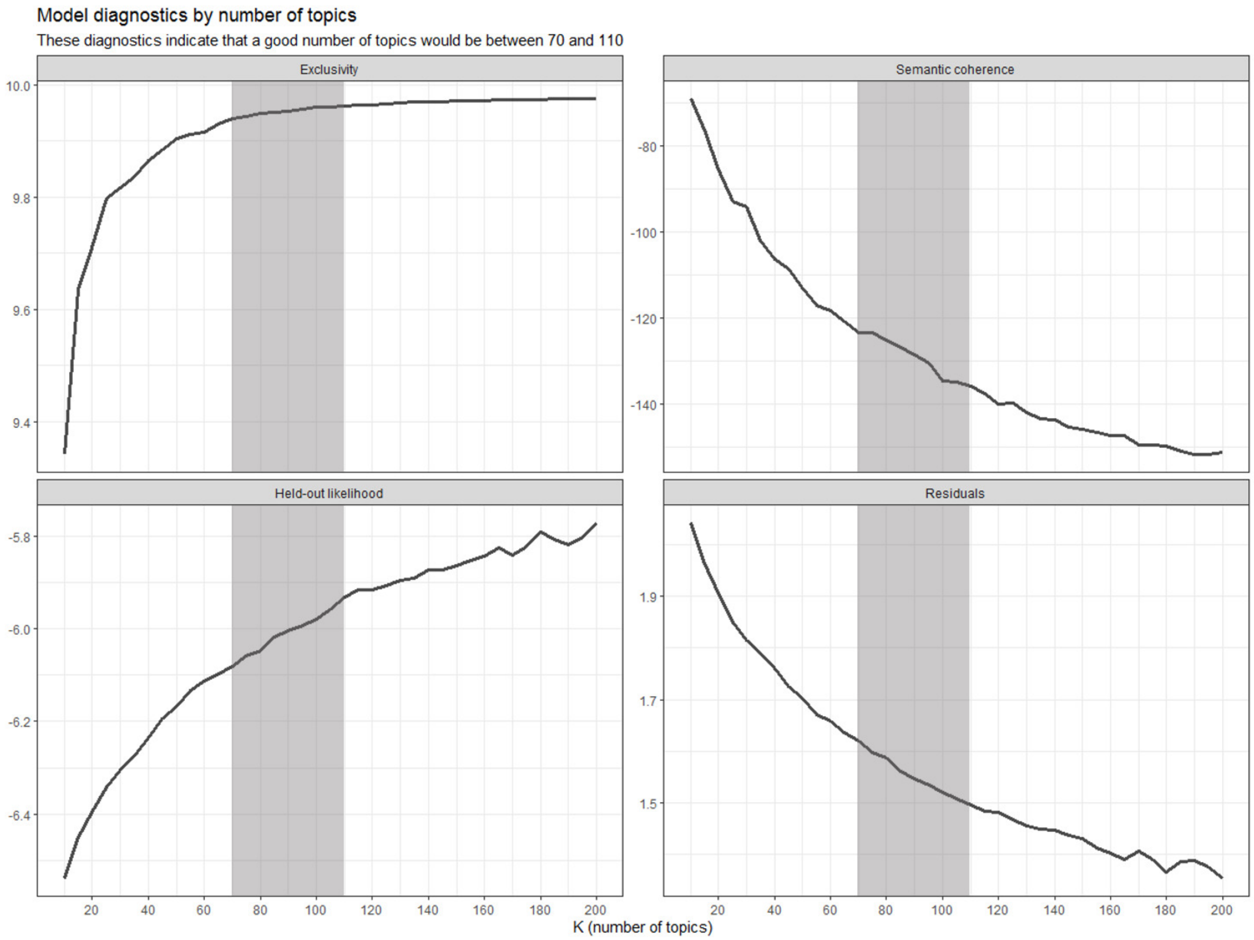

Figure S 2: Diagnostics of the STM as specified by Eq. 4 varying K from 5 to 200 in increments of 5. The shaded area represents the values of K we consider for the fit in the main text.

S 2 The U.S. labor market before and during the pandemic

In this section we discuss in more detail U.S. economic situation relevant for the Great Resignation. In particular, factors that stand out in the U.S. economy as plausible drivers of the Great Resignation.

One factor we discussed in the main text is the pro-cyclical behaviour of the quit rate, job openings and labor market tightness (i.e., the ratio between the number of job openings and unemployed workers). In addition to the quit rate shown in the main text, here in Figure S 3 (left) we show the pro-cyclicality of the job openings and labor market tightness of the U.S. economy. Although the pro-cyclicality likely played a role in the Great Resignation, none of the recession aftermaths since the 1990 created a similar surge in quits (Bureau of Labor Statistics, 2022*b*; Davis and Haltiwanger, 2014). During 2021, total quits and quits as a share of total separations reached a new record. Between 2001-2020 54% of all job separations were quits, in contrast, quits accounted for 69% of all job separations in 2021. In Figure S 3 (right) we show the relationship between the quit rate and job openings during recovery periods. We find that there has been a weakening between the quit and the hiring rate: in the twenty years prior to the pandemic, one additional job opening was associated with 0.38 additional job quits during recovery periods. In the COVID-19 recession recovery, an additional job opening has been associated with 0.29

quits.

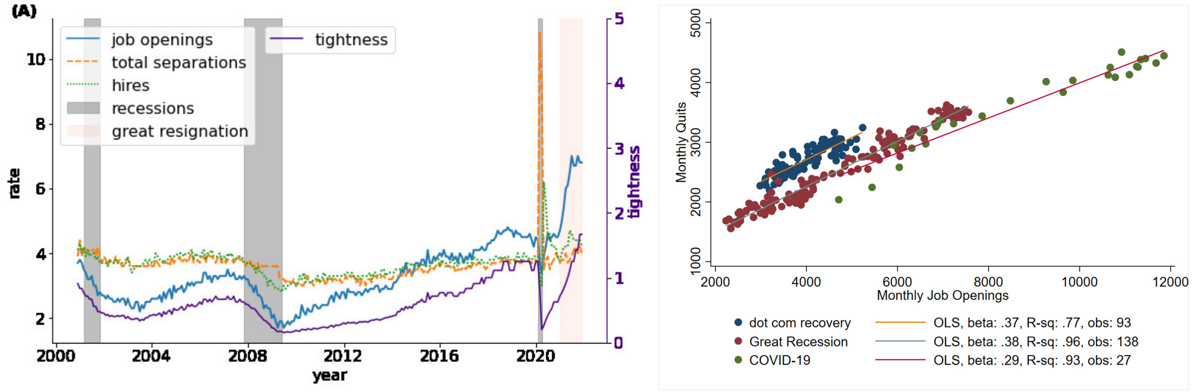

Figure S 3: **US labor market** Left (A). U.S. labor market statistics from December 2000 to December 2021. Recession periods are marked with shaded grey areas. The dashed line corresponds to March 2020 and the colored area to the Great Resignation period (year 2021). Right. The relationship between quits and job openings in recovery periods.

Instead of switching jobs, more people than ever seem to be opting into self-employment and start-ups (Mitchell and Dill, 2021). Furthermore, some of the registered quits may also be quits that people delayed or postponed during the pandemic (Cook, 2021). Another pull factor in the U.S. that in theory could have motivated people to quit was the stimulus policy enacted during the pandemic. However, Petrosky-Nadeau (2020) and Boar and Mongey (2020) show that these checks were too small and too temporary to make it worthwhile for people to quit in large numbers.

In the main text we discussed the push factors the pandemic unleashed and how these may have driven people to quit. Some of the effects of these push factors are recorded in moves to non-participation among older workers (Heggeness and Suri, 2021; Bureau of Labor Statistics, 2022*a*). After all, elderly workers were at disproportionate risk of suffering from COVID's health impacts. However, young people were also affected by the push factors through difficulties adapting to home office, struggles with daycare and school closures and burnout. As we show in Table 1, our sample of study is mostly young adults, therefore we most likely capture the effects of push factors not included in early retirement.

S 3 Reddit and work

In this section we discuss the subreddits 'r/jobs' and 'antiwork', and conclude that 'r/jobs' is better suited for our analysis. For 'r/jobs', the subreddit we focus our analysis on, we provide additional analysis on the dynamics.

S 3.1 'r/jobs' vs. 'r/antiwork'

To choose a subreddit suited for our study from 'r/jobs' and 'r/antiwork', we looked into i) the interests and ii) the posting activity across time of the users of both subreddits. We explored the useres' interests using the online tool "sayit" [6] , which computes Jaccard similarity between subreddits based on the subreddits where users comment. As shown in Figure S 4, although there is some overlap in the interest of users of these two subreddits, there are considerable differences. 'r/antiwork' is strongly political, and its users often post to other left-leaning (including openly "anticapitalist") political subreddits, such

[6] available online at https://github.com/anvaka/sayit

as ‘r/LateStageCapitalism’, ‘r/ABoringDystopia’, or ‘r/MurderedbyAOC’. In contrast, users posting to ‘r/jobs’ often post to other career-oriented subreddits, e.g., ‘r/AskHR’, ‘r/careerguidance’, or ‘r/resumes’. To examine the posting activity across time, we extracted all posts of both subreddits from 2016 to 2021. Table S 1 shows the raw number of posts (i.e., without filtering out spam and moderator posts) in each subreddit before and after the onset of the COVID-19 pandemic. Although ‘r/antiwork’ has more members than ‘r/jobs’, its popularity soared after the onset of the pandemic. In fact, less than 10% of the posts are before the pandemic. Instead, ‘r/jobs’ has more equal distribution of posts before and after March 2020.

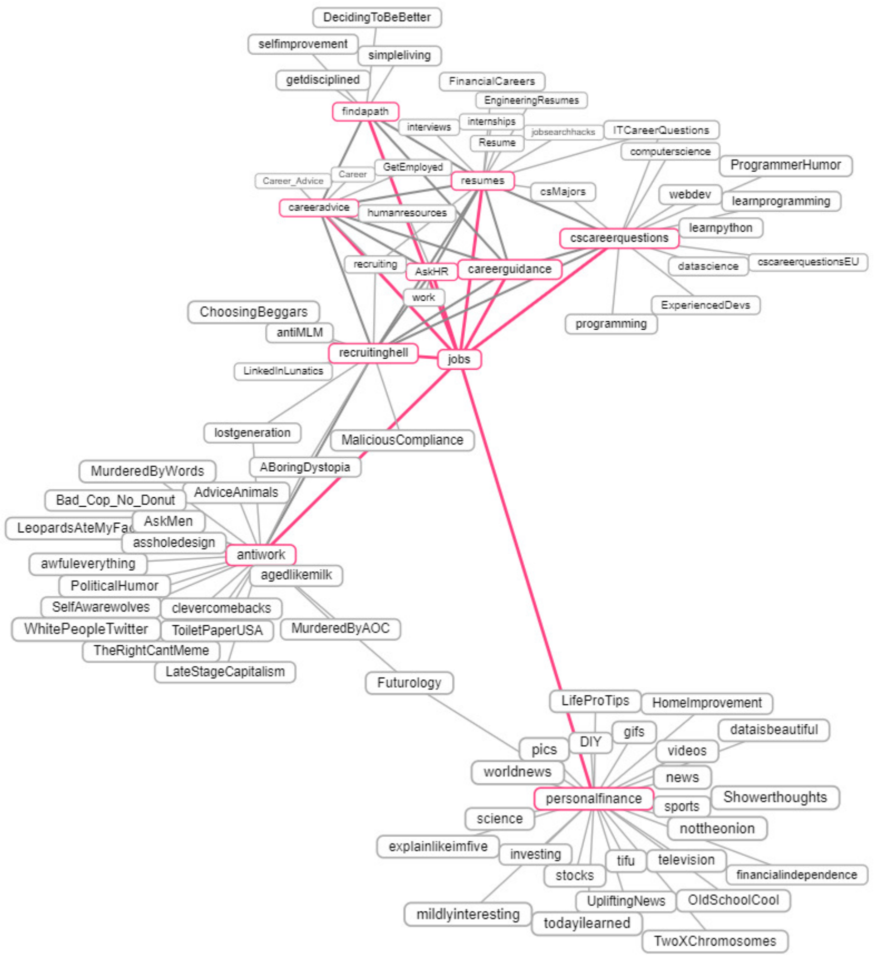

Figure S 4: **Co-occurrence of subreddit users** In this network, nodes represent subreddits, and ties denote that users who commented to subreddit A, also commented to subreddit B.

Since ‘r/jobs’ has a more balanced distribution of posts across time, and because its user composition is less politically skewed, we conclude that ‘r/jobs’ is a better subreddit to study the Great Resignation.

Measure	‘r/antiwork’	‘r/jobs’
Members (as of February 4th 2022)	1.5 million	0.65 million
Total number of posts (2018 - 2021)	134,212	269,647
Number of pre-pandemic posts (2018 - March 2020)	10,962	142,514

Table S 1: **Overview of work-related subreddits** Number of submissions from January 1st, 2018 to December 31st, 2021. These are the raw numbers of overall submissions and include some spam and moderator posts. For later analysis we filter several of these (see *Methods* section for details).

S 3.2 The dynamics of ‘r/jobs’

In this subsection, we provide additional information on the ‘r/jobs’ subreddit. Table S 2 shows the total number of posts from January 2018 and December 2021 in ‘r/jobs’ after filtering out spam, moderator posts, advertisements, etc. (see *Methods* section for details). Figure S 5 (left) shows the number of posts across years split by post with a flair, those without, and both. This figure shows a steady increase in the total number of posts in ‘r/jobs’. The number of posts has almost doubled between 2018 and 2021.

Measure (after filtering)	‘r/jobs’
Number of posts (2018-2021)	198081
Number of posts pre-Covid (2018- March 2020)	88092

Table S 2: **Number of posts in r/jobs.** These figures show the total number of ‘r/jobs’ after filtering.

Reddit allows users to add a tag to their posts to specify the content. For example, ‘r/baking’ allows *Recipe* and *No recipe* flairs to signal whether posts contain a recipe or not. Flairs are pre-defined, specific to each subreddit, and mutually exclusive, i.e., users select flairs from a limited number of available flairs, cannot create new ones, and can only use one flair per post. In our sample of ‘r/jobs’ posts there are 38 flairs (excluding those related to spam, moderation, etc.). The five most popular flairs (*Job searching*, *Interviews*, *Job offers*, *Leaving a job* and *Career planning*) account for roughly 50% of the flaired posts.

The bar plot in Figure S 5 (Right) shows the number of posts by year without a flair, with one of the top five flairs, or with one of the other flairs. This figure also shows the number of posts in each of the top five flairs, the most popular flair being *Job searching.* The name of the rest of the flairs are listed below:

1. ‘Applications’
2. ‘Article’
3. ‘Background check’
4. ‘Career planning’
5. ‘’Companies’
6. ‘Compensation’
7. ‘Contract work’
8. ‘Covid-19’
9. ‘Discipline’
10. ‘Education’
11. ‘Evaluations’
12. ‘HR’
13. ‘Internships’
14. ‘Interviews’
15. ‘Job offers’
16. ‘Job searching’
17. ‘Layoffs’
18. ‘Leaving a job’
19. ‘Networking’
20. ‘Office relations’
21. ‘Onboarding’
22. ‘Post-interview’
23. ‘Promotions’
24. ‘Qualifications’
25. ‘Recruiters’
26. ‘References’
27. ‘Rejections’
28. ‘Resumes/CVs’
29. ‘Startups’
30. ‘Temp work’
31. ‘Training’
32. ‘Unemployment’
33. ‘Work/Life balance’

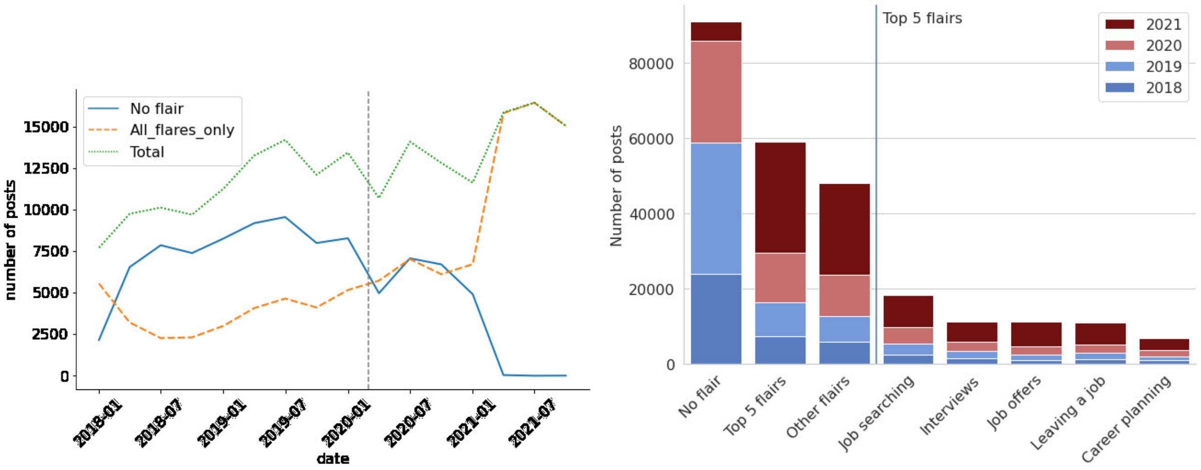

Figure S 5: **Distribution of r/jobs posts across time and flairs.** Figures show the total number of 'r/jobs' posts after filtering. Left: Total number of posts with and without flairs (2018-2021). Right: Total number of posts with no flair, top 5 flairs, and remaining flairs. Blue represents pre-pandemic years (2018 and 2019), red represents pandemic years (2020 and 2021).

To further validate whether 'r/jobs' reflects what happened in the U.S. labor market, we analyze how the popularity of different 'r/job' flairs developed over time. Since the five most popular flairs account for roughly 50% of the flaired posts we focus the overview on these flairs. As Figure S 6 shows, the popularity of the top five flairs (i.e. the share of posts corresponding to each flair) remained roughly constant until April 2021. In this month, which is also the month when the quit rate reached a record high in the U.S., there were some abrupt changes. *Job searching*'s popularity decreased sharply, while *Interviews* and *Leaving a job* increased their popularity. After April 2021 the share of the flairs did not reverse to the pre-pandemic trends. Instead *Job searching*' share remained lower than in pre-pandemic levels and the share of *Job offers* showed a steady increase.

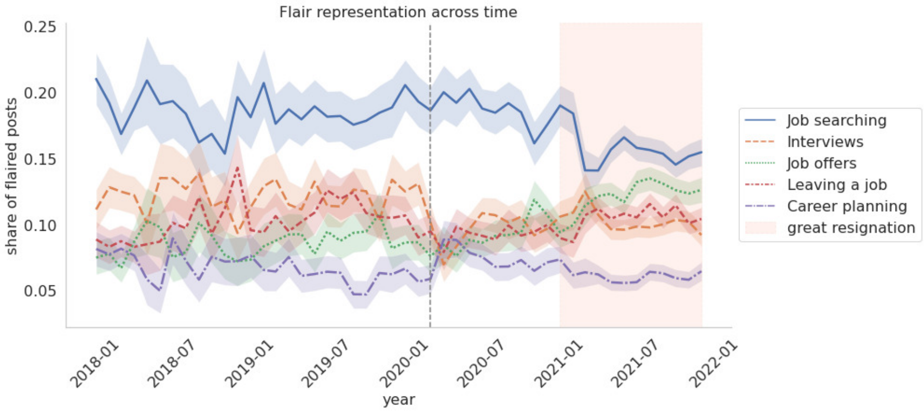

Figure S 6: **r/jobs posts across time.** This figure shows the share of posts tagged for each of the five most popular flairs. Approximately 50% of all posts are tagged with one of the top five flairs.

The average share of posts flaired under *Leaving a job* after April 2021 is roughly equal to the pre-pandemic average share. This finding might be surprising given the large number of quits that happened during the Great Resignation. One must bear in mind, however, that some posts flaired under *Leaving a job* may correspond to fires, while posts about quitting may have a different flair. For example, people

may mention they are quitting their job to take a different job and flair the post with *Job offers*. However, as we showed in the main text, when we distinguish posts as quit-related and fired-related, we find that the number of quit-related posts increased during the Great Resignation, while the fired-related posts spiked in the first months of the pandemic.

S 4 Sentiment analysis

Here we present additional results and robustness checks for the sentiment analysis across the whole sample of 'r/jobs' posts.

S 4.1 Sentiment before and during the pandemic and the Great Resignation

Did the sentiment around quitting change since the onset of the pandemic and the Great Resignation, and if so, in which direction? On the one hand, one could expect a better sentiment since unemployment benefits and COVID-19 stimulus payments may have created a cushion for those considering to quit. The increasing number of job vacancies may also have reduced concerns about having a job. On the other hand, the known pandemic challenges such as adapting to remote work, school closure, health issues, etc., may have worsened the sentiment around quitting a job. To answer this question we look at changes in the sentiment score of the 'r/jobs' posts. We use the NRC emotion lexicon (Mohammad and Turney, 2013), which identifies sentiment across polarity (positive and negative) and the eight basic emotions (fear, anger, sadness, disgust, joy, trust, surprise, and anticipation) according to the theory by Plutchik (2001). The NRC lexicon (Mohammad and Turney, 2013) has shown better performance in word-emotion lexicons than other approaches (Kušen et al., 2017).

We first explore the overall time trends of the NRC sentiments for the quit- and nonquit- related posts together (see Figure S 7). We find that in the two years leading to the pandemic the mood recorded on 'r/jobs' was deteriorating. Negative emotions such as fear, sadness and disgust were trending upwards, while joy and trust were downward trending. The early months of the pandemic show record high levels of negative, and record low levels of positive polarity. However, we also see a more recent reversal in these trends. Fear and anger have been declining since the beginning of 2021, coinciding with the Great Resignation period, while joy and trust have stabilized since mid 2020.

Next, we present a difference-in-differences analysis for the NRC polarity and emotion scores. We run this analysis using the specification provided by 2, but where y_i corresponds to a polarity or emotion. Figure S 8 shows the results for the difference-in-differences analysis for NRC polarity scores. The positive and negative polarity are noisy and do not show particularly interesting or significant trends. Therefore we focus most of the analysis on emotions.

Figure S 9 shows that negative emotions (fear, anger, sadness and disgust) all spiked among the quit-related posts relative to the control group in the 2nd quarter of 2020, but this effect was short-lived. We do not find any notable relative changes in emotions among quit-related posts since the start of the Great Resignation. This increase is short lived, and negative sentiment returns to pre-pandemic levels within two quarters after the start of the pandemic.

The quarter in which negative sentiment among job-quitters spiked is largely an outlier in terms of low quit rate, low rate of job openings, and high rate of layoffs in the U.S. economy (see Supplementary Materials S 2). Those quitting at this time, in the midst of the worst labor market they have experienced in their lifetime, seem to be quitting out of despair. Hence, our results suggest that at the start of the pandemic there was a wave of quits out of despair for a quarter of a year. Notwithstanding, as shown

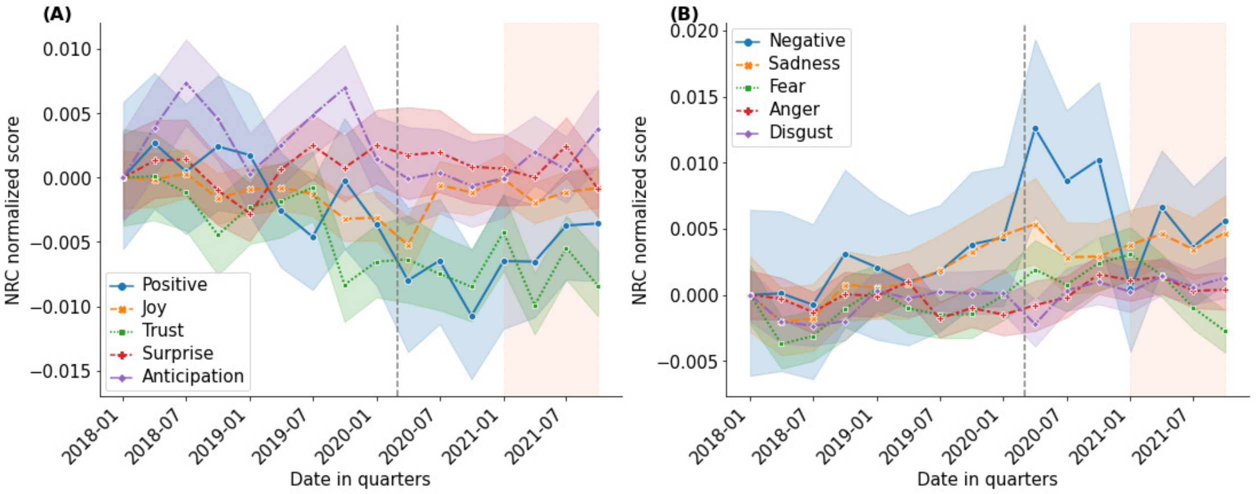

Figure S 7: **Sentiment analysis** This plot shows the dynamics of the sentiment score of 'r/jobs' posts. (A) Sentiments that are considered positive or neutral (B) Sentiments that are considered negative.

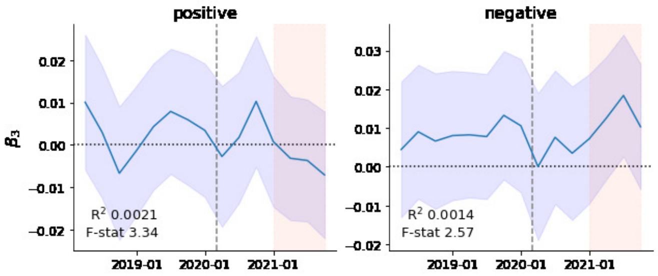

Figure S 8: Difference-in-Differences analysis between quit and non-quit posts. Results for positive and negative polarity using NRC.

by the U.S. quit rate, this wave had a much smaller number of quits relative to the numbers observed during the Great Resignation that followed.

Although the results obtained by sentiment analysis are informative and reveal how people quitting at the start of the pandemic were in despair, it is difficult to detect changes in narrative with this method. The sentiment analysis is a dictionary based approach agnostic to the topic being discussed. While it is useful to understand shifts in the emotional and sentiment load of texts, it would not detect changes in narratives when these are not accompanied by alterations in the emotions expressed. Furthermore, the topics change prevalence over time, and the changes in sentiment of each topic average out within the overall sentiment score. This is why our analysis in the main text relies heavily on the discourse in 'r/jobs' using STM.

We further clarify that we state no causal link between the sentiment analysis and the quits. There are many reasons why we cannot reach causality here. One is that contemplating quits is emotional too, and the relationship can be the other way around, from quits to emotions. One question that we think we can study in a causal way is how the emotional state around quit contemplations changed since the start of the pandemic and since the start of the Great Resignation, relative to the more general Reddit population (i.e., the control group). In particular, the timing of the sentiment changes between the two groups right around the start of the pandemic suggests that those quitting at the onset of the pandemic

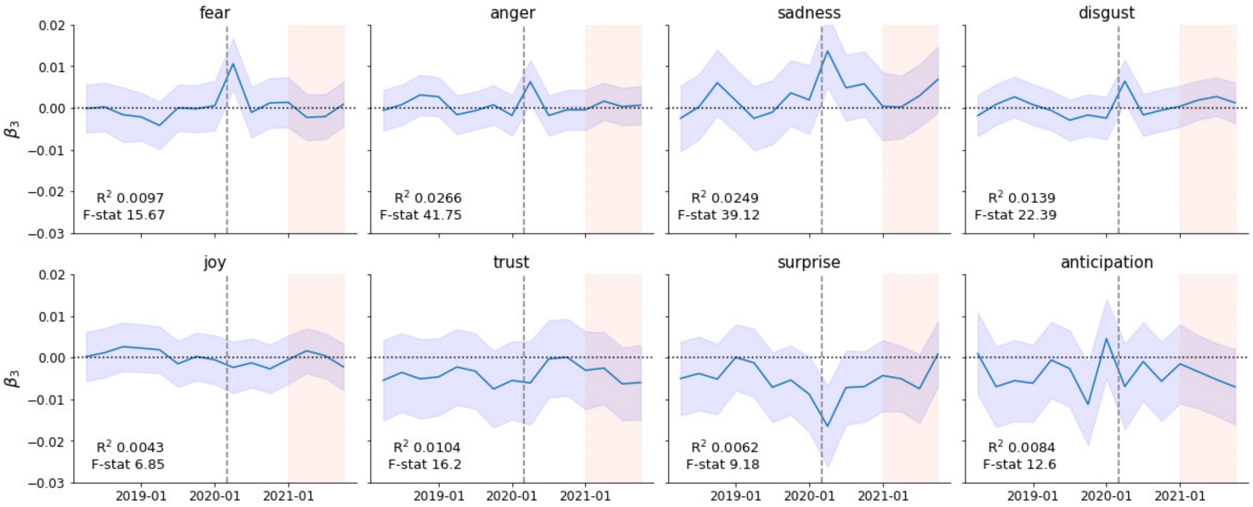

Figure S 9: **Sentiment analysis** This plot shows the interaction coefficients β_3 for each quarter. The vertical line corresponds to the start of the pandemic (March 2020) and the orange shaded area indicates the approximate period of the Great Resignation (year 2021). Below each sub-graph we show the adjusted R-squared and the F-stat of the regression model.

were discussing quitting while being particularly emotionally distressed.

S 4.2 Additional results using sentiment analysis

Sentiment across flairs To understand how the flair of a post relates to sentiment we look into the average sentiment for each of the top five flairs. We do this using all filtered posts and the NRC dictionary (Mohammad and Turney, 2013). We find that across the top five flairs Positive sentiment scores at the highest (see Figure S 10). This is somewhat surprising given that some flairs, such as *Leaving a job*, may be intuitively more linked with negative sentiment. Nonetheless, we find that the relative scores between posts of different flairs are intuitive. For example, posts flaired *Leaving a job* score high in Sadness, Anger, and Disgust. In contrast, *Job offers* posts score higher in Positive sentiment and lower in Fear. For the sentiment analysis in the subsection below we use a difference in differences approach that focuses on changes in sentiment scores before and after the pandemic. Since the NRC dictionary (Mohammad and Turney, 2013) captures relative differences between the flairs that are intuitive, we consider that the NRC dictionary (Mohammad and Turney, 2013) is an appropriate method to perform sentiment analysis. Nonetheless, for robustness, we look further into the NRC dictionary (Mohammad and Turney, 2013) below and use other sentiment analysis methods later in this appendix.

Understanding NRC results We looked further into what drove the results of NRC scores by looking at the posts with the highest negative scores. As the NRC gives a distribution of sentiments in each document, a negative score of one means only the negative sentiment was found. We took a random sample of 30 posts and looked closer at the words which triggered the negative score. Some of the words such as 'wrong' or 'tired' were coherent with our own expectations but some others, such as 'small', 'minimum', or 'foreign' were more questionable. Additionally, the word 'quit', particularly important in the present study, was classified as negative, which limits the possibility of quitting being considered as a positive and emancipating event (i.e., as described in Goldberg (2021)). We also looked into the words that were classified as positive. We find that both 'working' and 'job' are considered positive. These words explain why the predominant sentiment is positive. We do not think this hinders the results of our

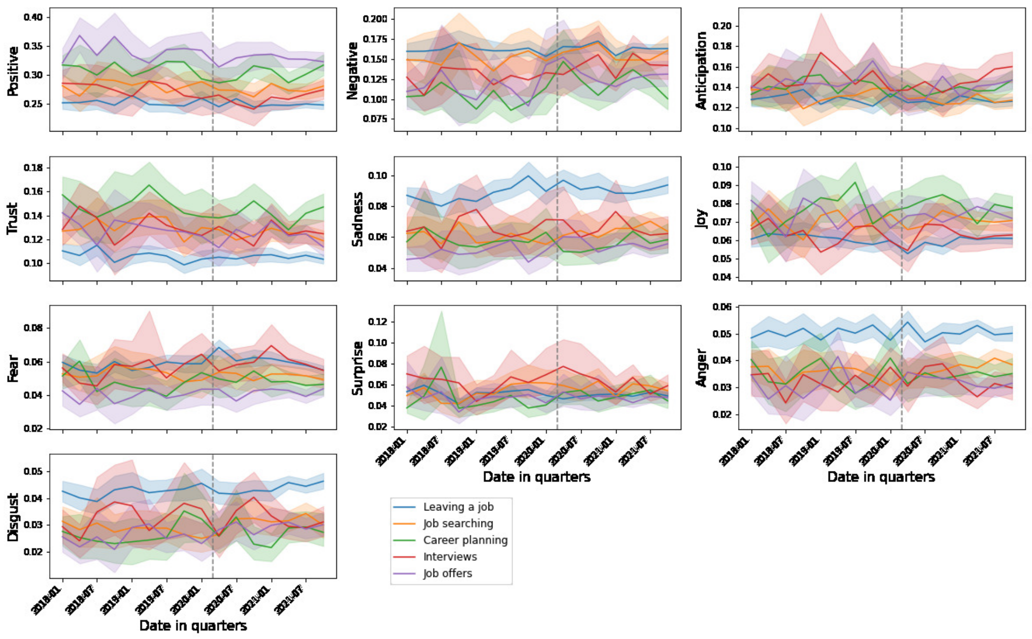

Figure S 10: **Sentiment analysis** This plot shows the average sentiment score across quarters for posts of different flairs. The grey line marks first of March 2020. Only posts from the quit and control group are included

analysis, since we focus on relative differences in sentiment before and after March 2020. Nonetheless, we run robustness checks with alternative sentiment methods.

S 4.3 Difference-in-differences analysis and alternative methods for sentiment analysis

Figure S 8 shows the results for the difference-in-differences analysis for NRC polarity scores. As discussed above, the positive and negative polarity are noisy and do not show particularly interesting or significant trends. To further validate our results we use two different sentiment analysis methods.

Different methods for sentiment analysis We use a different dictionary based approach, LIWC (Pennebaker et al., 2001). We measure the sentiment of the affective processes (positive emotion, negative emotion, anxiety, anger, and sadness) for the quit and control group and perform a difference in differences analysis as in the main text (see Figure S 11). Our results show that negative emotion and anxiety increased significantly among quit-related posts relative to non-quit posts during the first (and for anxiety also second) quarter of the pandemic. The rest of the sentiments did not show any significant change.

As a second robustness check on the sentiment analysis, we use VADER (Hutto and Gilbert, 2014), which is particularly developed for social media analysis. When using VADER we do not go through the pre-processing steps of lemmatization and negation handling, since VADER does not require these steps. The results are shown in Figure S 12. We see a significant increase in negative sentiment across quit-related posts relative to nonquit-related posts in the first two quarters of the pandemic. Once again,

this effect is not long-lasting, as negative sentiment returns back to pre-pandemic levels after the second quarter of the pandemic.

S 5 Topic modelling

In this section we present the results for all topics from our topic modelling analysis. Table S 4 shows the names of all topics and whether they are clear topics (CT), multi-topics (MT), or boiler-plate topics (BT). Figures S 13, S 14, and S 15 show the dynamics of the prevalence of all 90 topics across quit and non-quit related posts. Table S 5 shows the results of the difference in differences analysis for 78 interpretable topics. Moreover, in Figures S 16 and S 17 we present the mean sentiment for emotions across all topics that significantly changed their prevalence after the onset of the pandemic. In Table S 3 we report the point biserial correlation coefficients between sentiments and a dummy variable indicating whether a topic increased or decreased its prevalence after the onset pandemic. We did not find any evidence of relationship between the increase or decrease of a topic prevalence and the sentiment expressed.

Sentiment	Pearson-R	p-value
Fear	0.081	0.624
Anger	0.081	0.623
Sadness	0.215	0.188
Disgust	0.120	0.468
Joy	0.077	0.642
Trust	-0.304	0.060
Surprise	-0.146	0.375
Anticipation	-0.091	0.583
Positive	-0.052	0.754
Negative	0.156	0.342

Table S 3: **Correlation** Correlation between mean sentiment and dummy variable indicating whether a topic increased or decreased its prevalence after the onset pandemic.

Additional analysis on specific topics In the main text we also mention an increase in the prevalence of the multi-topic *health issues / healthcare job / scheduling* in quit-related posts with respect to non-quit posts. To try to understand which of the subtopics was driving the increase we also did a difference-in-differences analysis on different common words among the topic. We were not able to find a particular group of words to which we could clearly attribute the increase of the topic prevalence (see Figure S 18 for examples of words we looked into). As we conclude in the main text, we are not able to identify precisely what drives the increase of this topic and hence our results for this topic should be nuanced.

Table S 4: **Labels for the 90 topics and their keywords**. CL: clear topics; MT: multi-topics; BT: boiler-plate topics

Topic	Type	Raw label	Keywords
1	CL	Work-related distress	feel_like, feel, dont_feel, just_feel, feeling, lost, job_feel, like_just, completely, feeling_like
2	MT	seeking advice on quitting	quitting_job, unemployment, okay, short, half, text, paycheck, regret, possibly, story

Continued on next page

Table S 4 – continued from previous page

Topic	Type	Raw label	Keywords
3	CL	hate job & want to quit	quit_job, toxic, want_quit, just_quit, properly, toxic_work, workplace, quit_current, quit_dont, shitty
4	BT	"worth" / "soon"	worth, possible, considered, changing_job, soon, soon_possible, dilemma, promised, job_soon, considering
5	CL	switching job	pandemic, company_year, current_company, laid, working_company, employed, large, switching, large_company, switch_job
6	CL	remote jobs	remote, support, accounting, remotely, fully, country, remote_job, relocate, video, consultant
7	CL	job search	job_searching, searching, searching_job, nyc, unemployed, shot, actively, rough, turned, turning
8	CL	job opportunity	opportunity, great, growth, job_opportunity, grow, amazing, great_job, board, new_opportunity, awesome
9	BT	"need"	need_job, really_need, just_need, job_need, need_advice, need, dont_need, need_money, break, need_work
10	CL	guilt about leaving for a better job	leaving_job, better_job, feel_guilty, better, guilty, job_better, better_pay, burn, bridge, guilt
11	CL	make money	make, make_sure, want_make, make_money, money, sense, make_sense, sure, making, job_make
12	CL	salary negotiations	salary, bonus, raise, increase, higher, negotiate, base, range, lower, compensation
13	CL	work environment	want_work, work_environment, job_work, looking_work, like_work, place_work, work_just, environment, work, currently_work
14	CL	difficulty finding a job	finding, finding_job, hard_time, grad, hard, recent, difficult, trouble, having, harder
15	MT	got fired / got hired	just_got, got_job, got, job_got, got_fired, recently_got, got_hired, fired, finally_got, finally
16	CL	interview questions	job_interview, answer, interviewer, question, interview, answer_question, phone_interview, interview_tomorrow, second_interview, interview_job
17	CL	questions about giving two weeks notice	week_notice, notice, vacation, notice_period, week, giving, giving_notice, job_week, period, giving_week
18	CL	asking for advice	advice, advice_appreciated, thanks_advance, thanks, appreciate, appreciated, greatly_appreciated, advance, situation, job_advice
19	CL	background checks	check, background_check, background, record, employment, drug_test, paperwork, completed, credit, worried

Continued on next page

Table S 4 – continued from previous page

Topic	Type	Raw label	Keywords
20	CL	job hopping	stay, long, story_short, long_story_short, want_stay, long_term, staying, longer, term, long_time
21	CL	questions about specific job positions	position, current_position, offered_position, new_position, job_position, position_company, offered, position_year, different, open
22	CL	work schedule	day_week, day, work_day, weekend, shift, day_day, night, day_work, job_day, saturday
23	CL	leaving current job	leave_job, current_job, want_leave, leave, current, leave_current, just_leave, job_current, dont_want, want
24	CL	(resignation) letter	letter, resignation, signed, sign, resignation_letter, offer_letter, written, formal, write, recommendation
25	CL	job application process	application, job_application, address, applied_job, applicant, paper, form, number, applied, status
26	CL	career path	change, career, path, choice, career_path, industry, change_job, choose, goal, direction
27	CL	hating job	hate, absolutely, suck, miserable, dont_like, hate_job, everyday, tired, anymore, shit
28	CL	issues with white collars	team, project, workload, lead, member, leader, department, deadline, team_member, leadership
29	CL	recruitment companies	linkedin, recruiter, website, bank, profile, post, site, information, recruitment, recruiting
30	MT	trying to get a job / looking for a job for relatives	trying, figure, understand, dad, trying_job, foot, mom, door, trying_figure, dont_understand
31	CL	online job search	job_search, search, wife, realize, google, networking, network, resource, unique, connection
32	CL	job offer issues	job_offer, offer, offer_company, accepted, accepted_job, offer_job, accepting, got_offer, received_offer, accept
33	CL	interview rounds	land, round_interview, round, mcdonalds, final, land_job, managed, hard_work, work_hard, landed
34	BT	"look"	look_like, look_job, look_bad, look, job_look, make_look, like_job, look_good, job_job, eye
35	CL	hiring	hiring, hiring_manager, process, hiring_process, interview_process, candidate, stage, onboarding, red_flag, screening
36	BT	"good"	good_job, good, job_good, really_good, fit, good_fit, pretty_good, pretty, good_idea, think_good
37	MT	tech jobs / tests in hiring process	test, software, engineer, engineering, design, technical, learn, hotel, learning, technician
38	CL	commuting, moving for job	car, location, city, drive, driving, house, town, commute, mile, live

Continued on next page

Table S 4 – continued from previous page

Topic	Type	Raw label	Keywords
39	CL	seeking explanation of terms	doe, mean, sound, sound_like, anybody, director, doe_mean, usually, proceed, weird
40	BT	“right”	right, job_right, mess, hell, away, right_away, dumb, right_thing, sorry, idiot
41	BT	“changing”	changing, exact, jump, keep, switched, ground, catch, wonder, thing_just, thing
42	BT	“high”	paying, high, job_pay, bill, high_school, low, paying_job, decent, debt, pay
43	CL	clocking in late or early	late, early, met, meet, minute, fault, suppose, vent, missing, strange
44	CL	working from home	home, work_home, family, working_home, kid, disability, covid, child, baby, family_member
45	CL	new job	new_job, new, job_new, started_new, new_company, starting_new, looking_new, starting, got_new, start_new
46	CL	summer jobs and student internships	summer, internship, school, college, semester, class, college_student, intern, job_college, student
47	MT	quitting & paid time off / considering part time jobs	time_job, time, working_time, paid_time, work_time, time_time, waste, time_work, time_just, spend
48	CL	seeking advice on resumes	gap, resume, list, cover_letter, include, history, job_resume, work_history, explain, lie
49	CL	stocks, financial markets & equity	market, job_market, value, welcome, min, stock, share, heavily, option, labor
50	CL	working hours	hour_week, parttime, fulltime, hour, working_hour, parttime_job, work_hour, second_job, fulltime_job, hour_work
51	CL	college degree and job searching	field, bachelor, degree, bachelor_degree, job_field, graduated, master_degree, master, certification, education
52	CL	start date	reference, start, start_date, june, july, date, april, job_start, january, august
53	CL	job advice for people of age X	old, year_old, job_year, old_job, past_year, year, year_ago, worked_year, year_year, year_job
54	CL	management issues	employee, staff, nonprofit, force, organization, husband, rule, corporate, new_employee, management
55	MT	job postings / comparing two options / companies	job_posting, job_company, work_company, company_just, company_company, posting, big_company, company, company_want, company_work
56	CL	need help	help, need_help, thank, guy, reddit, hello, construction, hey, job_help, thank_advance
57	BT	business talk	business, people, lot_people, small_business, people_work, youre, like_people, usa, brand, arent

Continued on next page

Table S 4 – continued from previous page

Topic	Type	Raw label	Keywords
58	CL	entry level job issues	entry_level, entry_level_job, stuck, dead, entry_level_position, mid, progress, security, job_doing, level
59	CL	looking for tips (best ways how to)	whats, best, way, best_way, whats_best, approach, youve, suit, rep, worst
60	BT	"really" / "is it bad" questions	really_like, feel_bad, bad, really_want, really, job_really, really_bad, dont_really, bad_idea, idea
61	BT	"think"	quite, dont_think, think, sort, real, bit, essentially, somewhat, kind, probably
62	CL	online jobs to make extra money	free, marketing, earn, amazon, teacher, teaching, online, teach, writing, social_media
63	BT	uncertainty about life / jobs	dont_know, know, want_know, know_job, let_know, let, want_job, job_know, job_dont, know_just
64	CL	not meeting work experience required by job ads	work_experience, year_experience, experience, skill, relevant, qualification, volunteer, job_experience, lack, require
65	CL	quit	quit, job_quit, today, job_today, spot, quit_week, quit_just, scared, understaffed, wanting
66	CL	dream job	dream, dream_job, love, wanted, rejected, loved, just_wanted, love_job, wanted_work, really_wanted
67	CL	job titles, promotions	title, role, description, job_title, responsibility, job_description, promotion, title_say, new_role, current_role
68	CL	follow-up emails or calls	follow, sent, email, emailed, monday, heard, friday, saying, havent_heard, phone
69	CL	blue collar and service job issues	warehouse, owner, order, food, worker, service, cleaning, kitchen, server, table
70	CL	office jobs issues	office, office_job, admin, desk, sit, assistant, receptionist, sitting, space, duty
71	CL	worries about employer while job searching	leaving, employer, previous, previous_job, reason, left_job, potential_employer, left, current_employer, previous_employer
72	CL	retail job issues	retail, store, retail_job, fast_food, customer, restaurant, cashier, seasonal, grocery_store, customer_service
73	CL	quitting a new job	quitting, just_started, started, started_working, thinking, thinking_quitting, job_just, recently_started, started_job, job_started
74	CL	reviews of workers and companies	review, performance, negative, positive, exam, feedback, poor, probation, improve, glassdoor
75	CL	issues involving boss	bos, shes, boss, tell, tell_bos, told_bos, upset, current_bos, coworkers, bos_said

Continued on next page

Table S 4 – continued from previous page

Topic	Type	Raw label	Keywords
76	CL	issues involving a manager	rant, manager, assistant_manager, new_manager, promoted, manager_told, manager_said, general, told_manager, manager_position
77	CL	job hunting	hunting, job_hunting, hunt, job_hunt, rejection, wasnt, march, start_job, luckily, decided
78	CL	university related questions	program, university, study, research, finance, international, studying, uni, phd, math
79	CL	asking for advice related to situation X	didnt, said, wasnt, knew, told, kept, called, asked, didnt_want, came
80	CL	mental health	anxiety, mental_health, lined, stress, job_lined, depression, mental, worse, physical, mentally
81	CL	issues with coworkers	supervisor, coworker, meeting, woman, mistake, group, colleague, task, uncomfortable, talk
82	MT	tech companies / small vs large companies	switch, tech, firm, small_company, consulting, pro, con, small, tech_company, smaller
83	CL	resigning	resign, concern, responsible, case, claim, terminated, forced, agreement, resigned, termination
84	MT	health issues / jobs in healthcare / scheduling issues	hospital, medical, patient, doctor, appointment, scheduling, request, requested, facility, healthcare
85	CL	issues related to contracts	ceo, agency, client, contractor, insurance, contract, experienced, package, agent, payroll
86	CL	applying for jobs	applying, applying_job, apply, getting_job, chance, apply_job, job_applying, gotten, getting, chance_getting
87	BT	"month"	month_ago, job_month, couple_month, month, quit_month, past_month, ago, month_just, month_later, month_month
88	CL	temp jobs	hire, training, temp, hired, train, week_ago, temporary, couple_week, permanent, new_hire
89	MT	looking for jobs / sales jobs related questions	looking_job, sale, looking, suggestion, start_looking, job_looking, startup, sell, just_looking, product
90	MT	asking for advice (broad)	regardless, going, ill, turn, basically, come, thought, wont, there, actually

Table S 5: **Difference in differences analysis for the 78 interpretable topics**. CL: clear topics; MT: multi-topics; and BT: boiler plate topics

Topic	Type	Label	Parallel trends?	R-squared	F-statistic
1	CL	work-related distress	yes	0.027	46.3
2	MT	seeking advice on quitting	yes	0.051	92.19
3	CL	hate job & want to quit	no	0.173	372.24
5	CL	switching job	nearly yes	0.028	46.43

Continued on next page

Table S 5 – continued from previous page

Topic	Type	Label	Parallel trends?	R-squared	F-statistic
6	CL	remote jobs	yes	0.034	57.54
7	CL	job searching	nearly yes	0.007	13.09
8	CL	job opportunity	no	0.002	4.24
10	CL	guilt about leaving for a better job	nearly yes	0.058	112.17
11	CL	make money	no	0	1.55
12	CL	salary negotiations	no	0.002	4.12
13	CL	work environment	no	0.007	13.49
14	CL	difficulty finding a job	yes	0.007	13.6
15	MT	got fired/got hired	no	0.002	4.25
16	CL	interview questions	no	0.085	156.65
17	CL	questions about giving two weeks notice	no	0.094	182.81
18	CL	asking for advice	yes	0.029	53.24
19	CL	background checks	no	0.003	8.42
20	CL	job hopping	yes	0.045	81.15
21	CL	questions about specific job positions	yes	0.014	25.38
22	CL	work schedule	no	0.025	44.3
23	CL	leaving current job	yes	0.122	239.57
24	CL	resignation letter	yes	0.012	20.79
25	CL	job application process	nearly yes	0.056	102.62
26	CL	changing career path	no	0.007	12.27
27	CL	hating job	no	0.051	93.33
28	CL	issues with white collars	yes	0.005	9.24
29	CL	recruitment companies	no	0.085	159.28
30	MT	trying to get a job / looking for a job for relatives	yes	0.007	13.35
31	CL	online job search	yes	0.014	26.71
32	CL	job offer issues	yes	0.002	3.77
33	CL	interview rounds	yes	0.019	28.78
35	CL	hiring	nearly yes	0.038	68.8
37	MT	tech jobs*	no	0.042	75.09
38	CL	commuting, moving for job	yes	0.003	7.43
39	CL	seeking explanation of terms	yes	0.053	96.86
43	CL	clocking in late/early	yes	0.001	3.22
44	CL	working from home	yes	0.024	41.9
45	CL	new job	no	0.04	74.32
46	CL	summer jobs / internships for college students	nearly yes	0.017	28.29
47	MT	quitting & paid time off — considering part time jobs	no	0.008	14.96

Continued on next page

Table S 5 – continued from previous page

Topic	Type	Label	Parallel trends?	R-squared	F-statistic
48	CL	seeking advice on resumes	yes	0.021	37.92
49	CL	stocks, financial markets & equity	no	0.006	11.4
50	CL	working hours	no	0.005	10.85
51	CL	college degree and job searching	no	0.033	56.7
52	CL	start date	yes	0.005	9.3
53	CL	job advice for people of age X	no	0.005	10.01
54	CL	management issues	no	0.012	22.05
55	MT	job postings*	no	0.005	10.21
56	CL	need help	no	0.058	111.22
58	CL	entry level job issues	no	0.008	15.4
59	CL	looking for tips (best ways how to)	yes	0.004	8.59
62	CL	online jobs/ extra money	yes	0.051	91.83
64	CL	not meeting work experience required by job ads	no	0.075	139.67
65	CL	quit	no	0.325	834.01
66	CL	dream job	no	0.001	2.92
67	CL	job titles, promotions	no	0.005	9.26
68	CL	follow-up emails/calls	yes	0.046	79.24
69	CL	blue collar/service job issues	no	0.002	4.63
70	CL	office jobs issues	no	0.001	2.51
71	CL	worries related to employer while job searching	nearly yes	0.063	114.41
72	CL	retail job issues	nearly yes	0.001	3.06
73	CL	quitting a new job	nearly yes	0.14	274.59
74	CL	performance review	no	0.002	4.59
75	CL	issues involving boss	no	0.069	132.2
76	CL	issues involving a manager	no	0.008	13.96
77	CL	job hunting	yes	0.001	3.49
78	CL	university related questions	no	0.043	77.05
79	CL	asking for advice related to situation X	yes	0.014	25.11
80	CL	mental health	nearly yes	0.064	113.24
81	CL	issues with coworkers	no	0.009	16.39
82	MT	tech companies*	yes	0.002	3.84
83	CL	resigning	no	0.042	77.94
84	MT	health issues*	yes	0.001	1.74
85	CL	issues related to contracts	yes	0	1.76
86	CL	applying for jobs	nearly yes	0.048	87.88

Continued on next page

Table S 5 – continued from previous page

Topic	Type	Label	Parallel trends?	R-squared	F-statistic
88	CL	temp jobs	yes	0.008	15.81
89	MT	looking for jobs*	yes	0.045	84.63
90	MT	asking for advice	yes	0.019	31.21

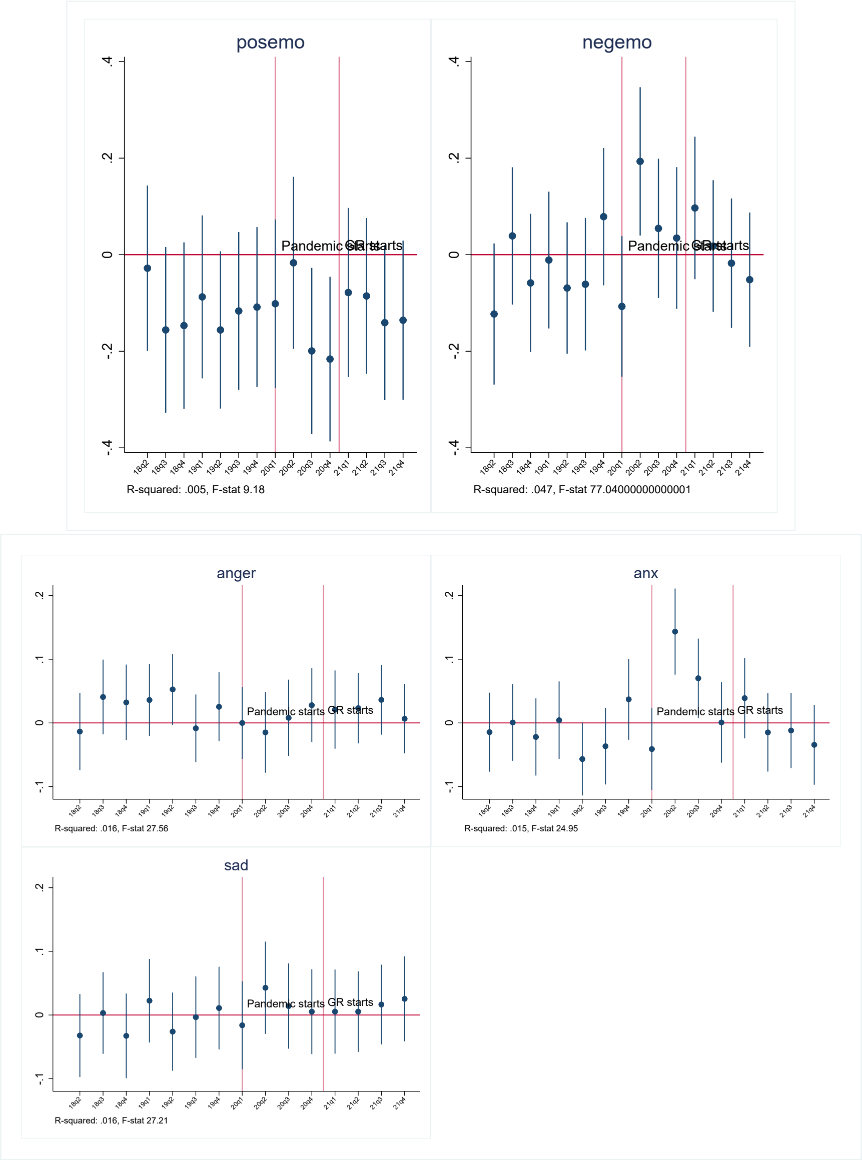

Figure S 11: **LIWC sentiment analysis** Difference in differences analysis for positive and negative emotion (top panels) and anxiety, anger and sadness (bottom panels). The first red line from left to right marks the start of the pandemic, the second red line marks the Great Resignation period starting

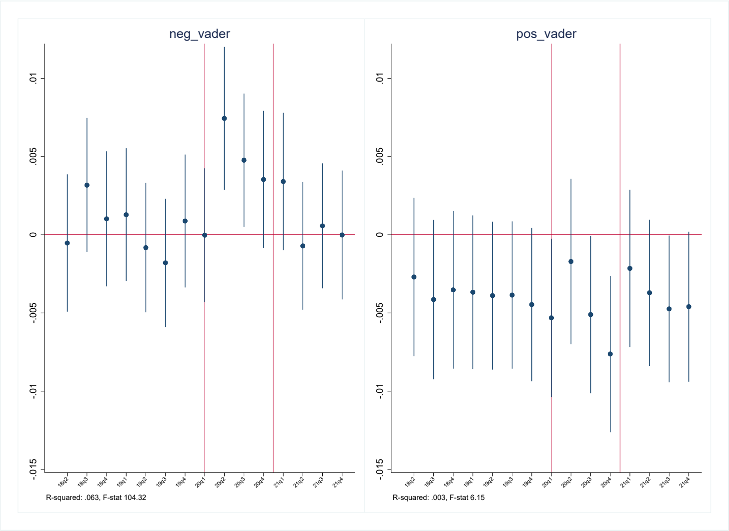

Figure S 12: **Vader sentiment analysis** Difference in differences analysis for positive and negative sentiment. The first red line from left to right marks the start of the pandemic, the second red line marks the Great Resignation period starting

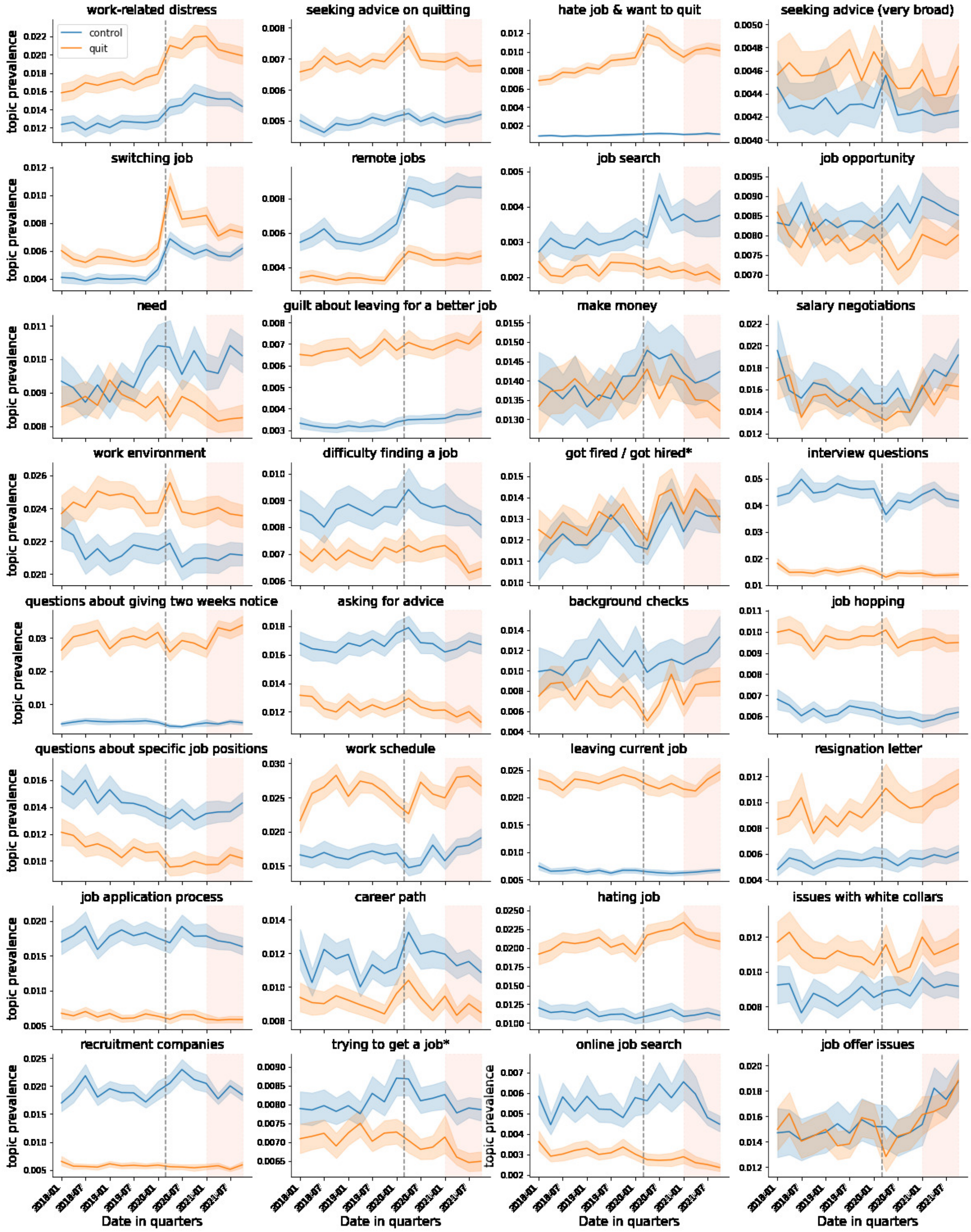

Figure S 13: **Topics prevalence** This plot shows the dynamics of the prevalence of different topics

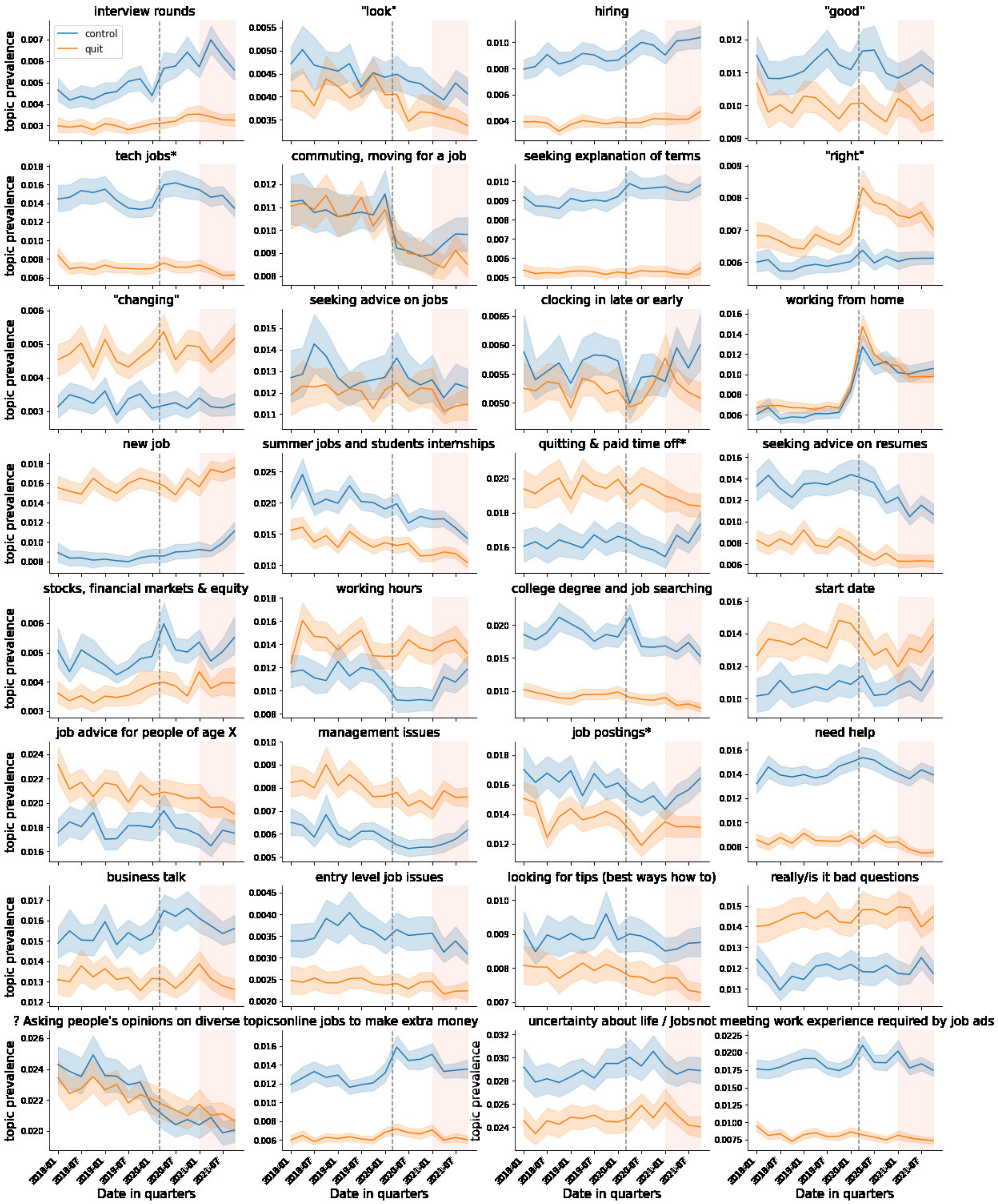

Figure S 14: **Topics prevalence** This plot shows the dynamics of the prevalence of different topics

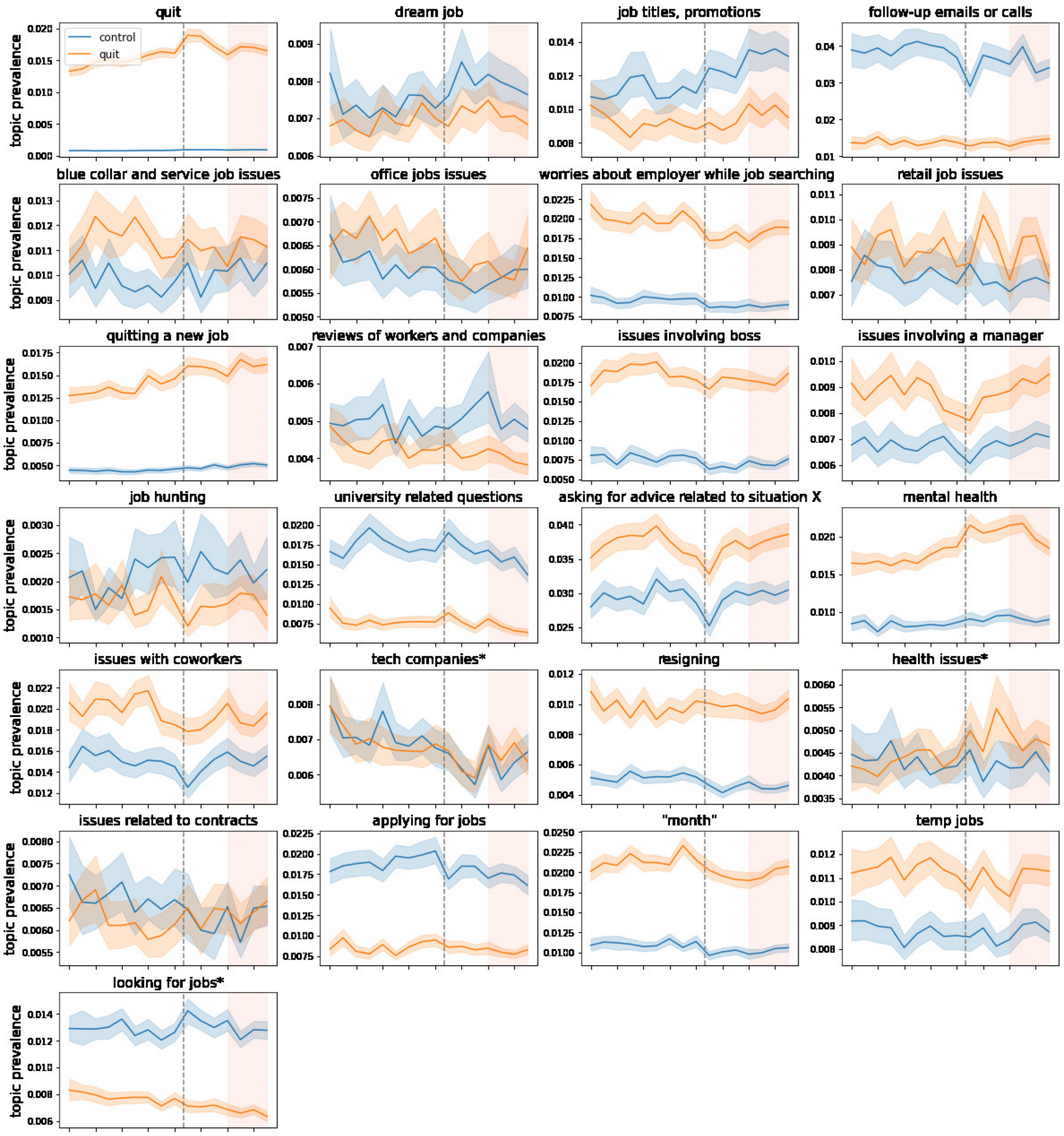

Figure S 15: **Topics prevalence** This plot shows the dynamics of the prevalence of different topics

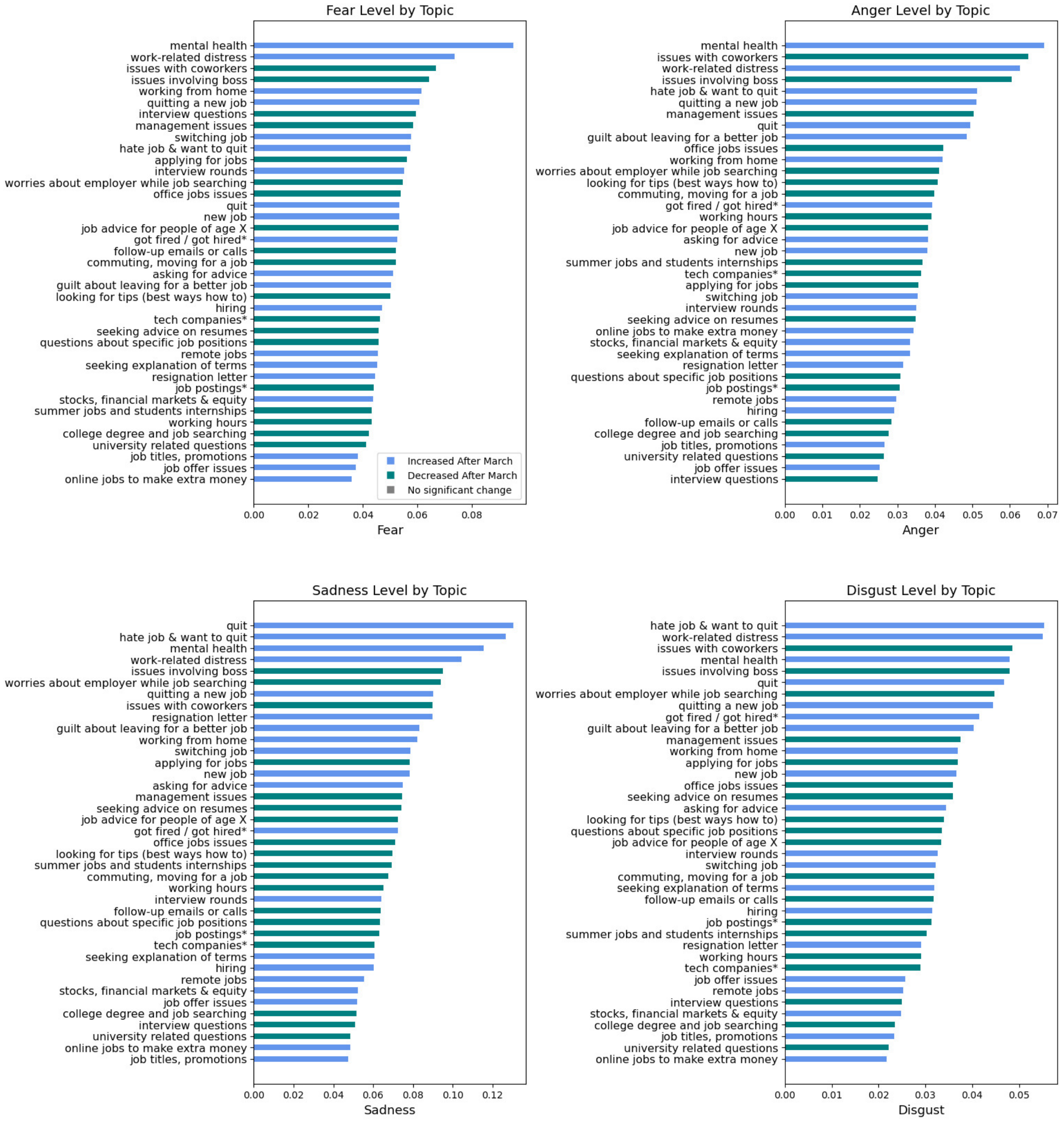

Figure S 16: **Topics' sentiment** This plot shows emotions across all topics that significantly changed their prevalence after the onset of the pandemic

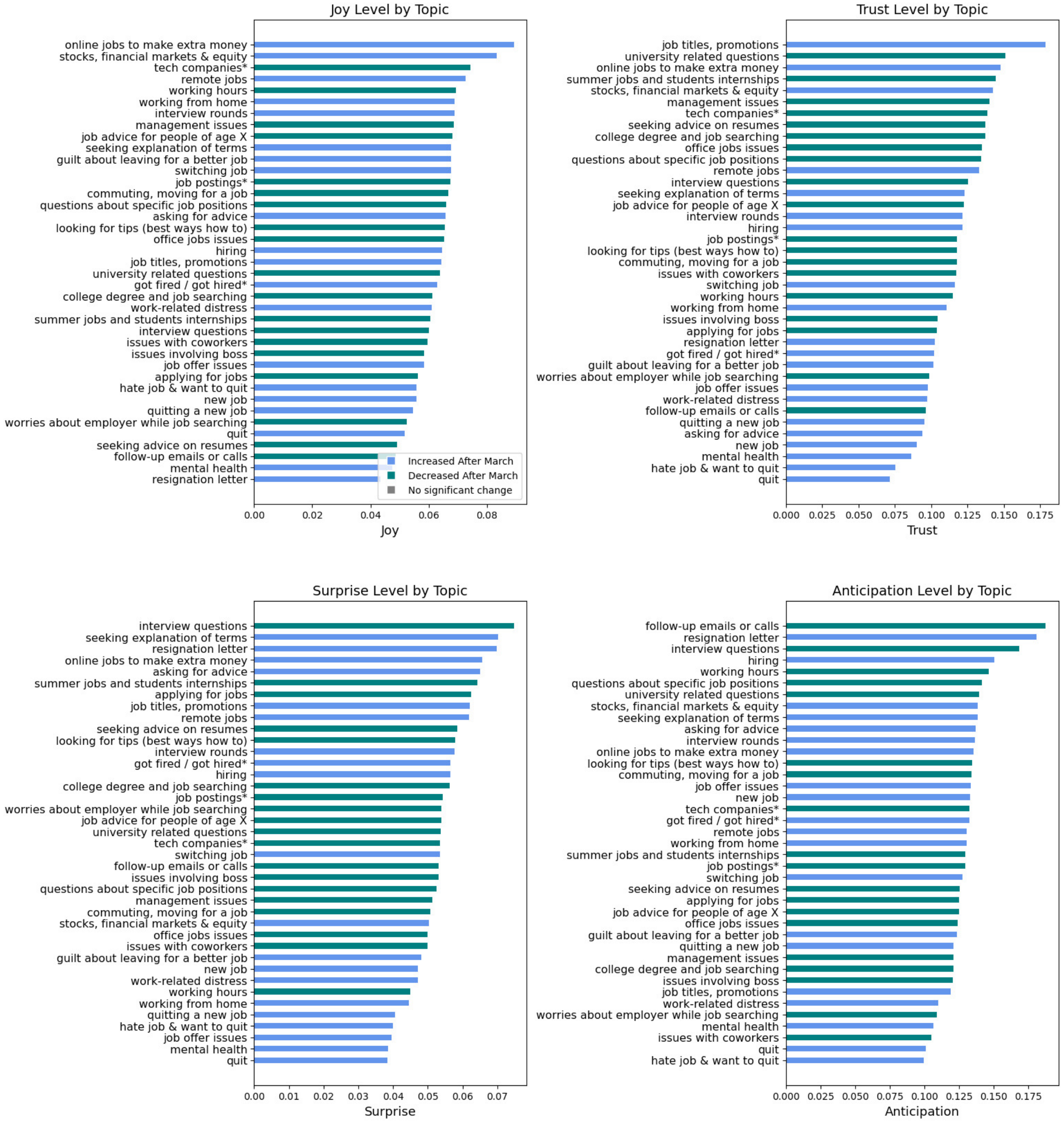

Figure S 17: **Topics' sentiment** This plot shows emotions across all topics that significantly changed their prevalence after the onset of the pandemic

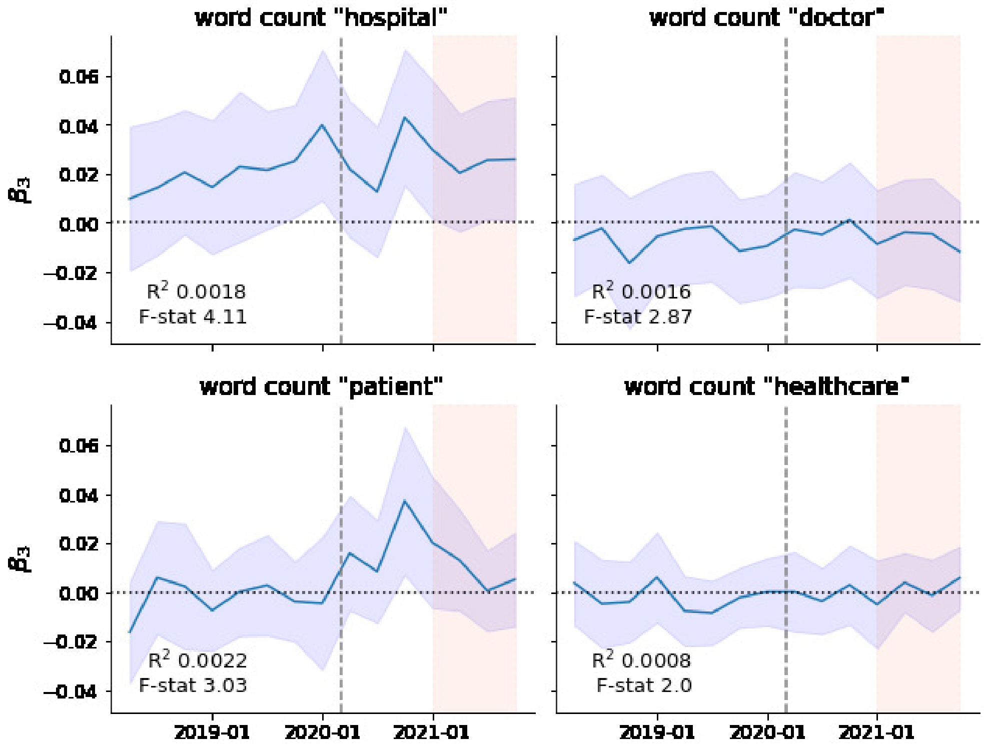

Figure S 18: Difference-in-differences analysis on the different common words among the topic “hate job & want to quit”
